# Efficient Dual Cas9 Nickase Correction of a Prevalent Pathogenic *LAMB3* Variant for Junctional Epidermolysis Bullosa

**DOI:** 10.1016/j.xjidi.2024.100343

**Published:** 2024-12-24

**Authors:** Alex du Rand, John Hunt, Daniel Verdon, Ben Buttle, P. Rod Dunbar, Diana Purvis, Vaughan Feisst, Hilary Sheppard

**Affiliations:** 1 School of Biological Sciences, The University of Auckland, Auckland, New Zealand; 2 Maurice Wilkins Centre, The University of Auckland, Auckland, New Zealand; 3 Te Whatu Ora Health New Zealand, Te Toka Tumai, Auckland, New Zealand

**Keywords:** CRISPR/Cas9, Gene correction, Gene therapy, Homology-directed repair, Skin grafts

## Abstract

Gene editing facilitated by homology-directed repair represents a promising strategy for precisely correcting pathogenic variants underlying monogenic disorders, including the life-threatening skin blistering condition junctional epidermolysis bullosa (JEB). Frequent reports of unintended off-target genotoxicity associated with conventional Cas9 nuclease editing have increasingly led to the adoption of dual-Cas9 nickases (dual-Cas9n) owing to their improved safety profile. However, rates of precise repair obtained with such strategies remain low. In this study, we establish a dual-Cas9n approach targeting *LAMB3*, using electroporation to deliver Cas9-nickase ribonucleoproteins and modified single-stranded oligodeoxynucleotide repair templates into primary JEB keratinocytes. Targeting a hotspot pathogenic variant (c.1903C>T, p.R635∗), we report perfect correction efficiencies of up to 54% based on standard next-generation sequencing. Using a high-fidelity Cas9 nuclease, we also report perfect repair of up to 74% when using a small-molecule modulator of DNA repair. Dual-Cas9n–corrected JEB keratinocytes demonstrated restored laminin-332 expression and secretion *in vitro*, leading to improved cellular adhesion and accurate laminin-332 localization in engineered skin equivalents. This protocol represents a significant improvement in precision gene repair using Cas9 nickases for epidermolysis bullosa, with the potential to be applied to a large cohort of patients harboring this prevalent pathogenic variant.

## Graphical abstract

**Figure:**
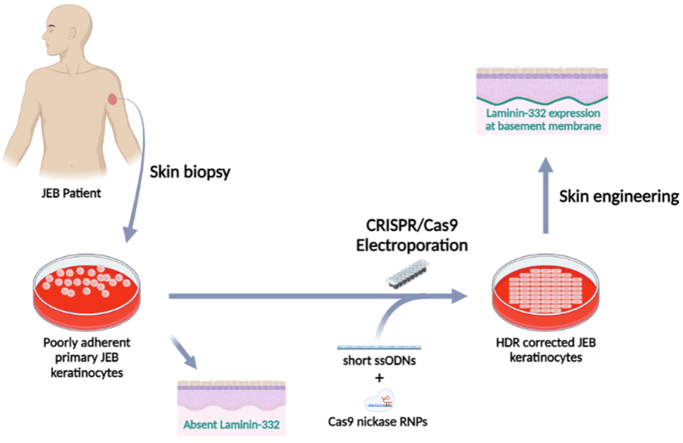

## Introduction

Epidermolysis bullosa (EB) is a rare, inherited group of heterogenous dermatoses characterized by fragility and blistering of the mucocutaneous membranes (Bischof et al, 2024). The junctional EB (JEB) subtypes are primarily caused by biallelic pathogenic variants in the laminin-332 heterotrimer (*LAMA3*, *LAMB3*, *LAMC2*), critical structures that anchor basal keratinocytes to the basement membrane (Kiritsi et al, 2013). Pathogenic *LAMB3* variants account for approximately 70% of all reported cases (Kiritsi et al, 2013) and disrupt the structural integrity of anchoring filaments, leading to dermal–epidermal skin fragility and blistering at the level of the lamina lucida. Large chronic skin wounds can significantly diminish the quality of life for patients, result in persistent scarring and infection, and increase susceptibility to aggressive skin cancers (du Rand et al, 2022). There is currently no curative therapy for JEB, and >40% of patients do not survive beyond adolescence (Fine et al, 2008).

The accessibility of human skin tissue has made EB an increasingly explored target for gene therapy (Bischof et al, 2024). To date, ex vivo gene replacement based on the retroviral-mediated addition of wild-type *LAMB3* has proven the most effective strategy for JEB. Several clinical studies have reported remarkable long-term results using this approach (Bauer et al, 2017; Hirsch et al, 2017; Mavilio et al, 2006), which has limited exploration of alternative gene therapy strategies at the *LAMB3* locus. CRISPR/Cas9-based gene editing represents a potentially promising alternative because it can directly target pathogenic variants, bypassing the need for retroviral vectors which rely on semirandom integration into the genome (Biasco et al., 2012; Hirsch et al, 2017). In addition, gene editing can address both dominant and recessive variants and enable restoration of physiological gene expression by maintaining the endogenous transcriptional regulation of the cell. Using this approach, a programmable single-guide RNA (sgRNA) can target a Cas9 nuclease to specific sites in the genome to generate double-strand breaks (DSBs). Mutagenic end-joining repair (including nonhomologous end-joining [NHEJ] and alternative end-joining) constitutes the dominant repair mechanism and can be used to reframe pathogenic variants or to delete faulty exons (Bonafont et al, 2019; Takashima et al, 2019). These approaches are efficient but can generate a high frequency of unpredictable insertions and deletions (INDELs) and are unable to address all pathogenic variants. A more favorable strategy is precise repair using the high-fidelity homology-directed repair (HDR) pathway, which can be promoted through the inclusion of a donor DNA repair template (Nambiar et al, 2022). Precise repair of any pathogenic variant remains the ultimate gene editing goal of EB and could theoretically be accomplished by HDR-mediated approaches. However, further improvements in editing efficacy are necessary before such approaches can be fully realized.

Therapeutic gene editing for EB using Cas9 nuclease is currently limited by frequent reports of off-target genotoxicity (Benati et al, 2018; Bischof et al, 2022; Klermund et al, 2024; Kocher et al, 2017, 2021; Petković et al, 2022; Webber et al, 2016). To address this risk, the catalytically modified Cas9 nickase variant can be employed to generate single-stranded DNA ‘nicks.’ When dual Cas9 nickases (dual-Cas9n) are used in close proximity, both strands can be nicked to generate a staggered DSB substrate for HDR repair (Nambiar et al, 2022). Several studies targeting EB-related genes have demonstrated that Cas9 nuclease off-target activity can be ablated when using the same gRNAs in combination with dual-Cas9n (Bischof et al, 2022; Kocher et al, 2021; Petković et al, 2022; Webber et al, 2016). Although promising, the facilitation of HDR by dual-Cas9n has generally been lower than by Cas9 nuclease. A promising study by Kocher et al (2021) reported up to 21% HDR in primary recessive dystrophic EB (RDEB) keratinocytes targeting *COL7A1*. Nevertheless, higher correction efficiencies are likely necessary to ensure sufficient restoration of functional protein and to routinely target enough epidermal stem cells (EpiSCs) (Kern et al, 2009; Schwieger-Briel et al, 2015).

Numerous reports of Cas9-based gene editing targeting other causative EB genes have been published (namely *COL7A1* for RDEB) (reviewed by Bischof et al [2024]). To the best of our knowledge, only a single gene editing study has been described targeting *LAMB3* (Benati et al, 2018). In this study, adenovectors carrying Cas9 nuclease and a sgRNA in combination with lentiviral vectors harboring the entire *LAMB3* cDNA were used to target a pathogenic splice site variant in intron 2. Low levels (0.5%) of HDR were achieved, with off-target nuclease activity (0.9%) reported at 1 analyzed off-target site (Benati et al, 2018).

To improve both the efficiency and safety of precise HDR repair targeting *LAMB3*, we established a retroviral-free, dual-Cas9n correction strategy targeting the most prevalent pathogenic variant (c.1903C>T, p.R635∗). Implementation of this strategy in bulk primary JEB keratinocytes enabled highly efficient and precise HDR repair, resulting in rescue of keratinocyte adhesion in vitro and restoration of laminin-332 expression in engineered skin equivalents (SEs). These data represent a significant improvement in the rates of precise gene repair for EB, opening the door for therapeutic gene editing facilitated by Cas9 nickases.

## Results

### Dual-Cas9 nickase efficiently repairs a prevalent pathogenic *LAMB3* variant (c.1903C>T) in primary JEB keratinocytes with high fidelity

**Figure 1:**
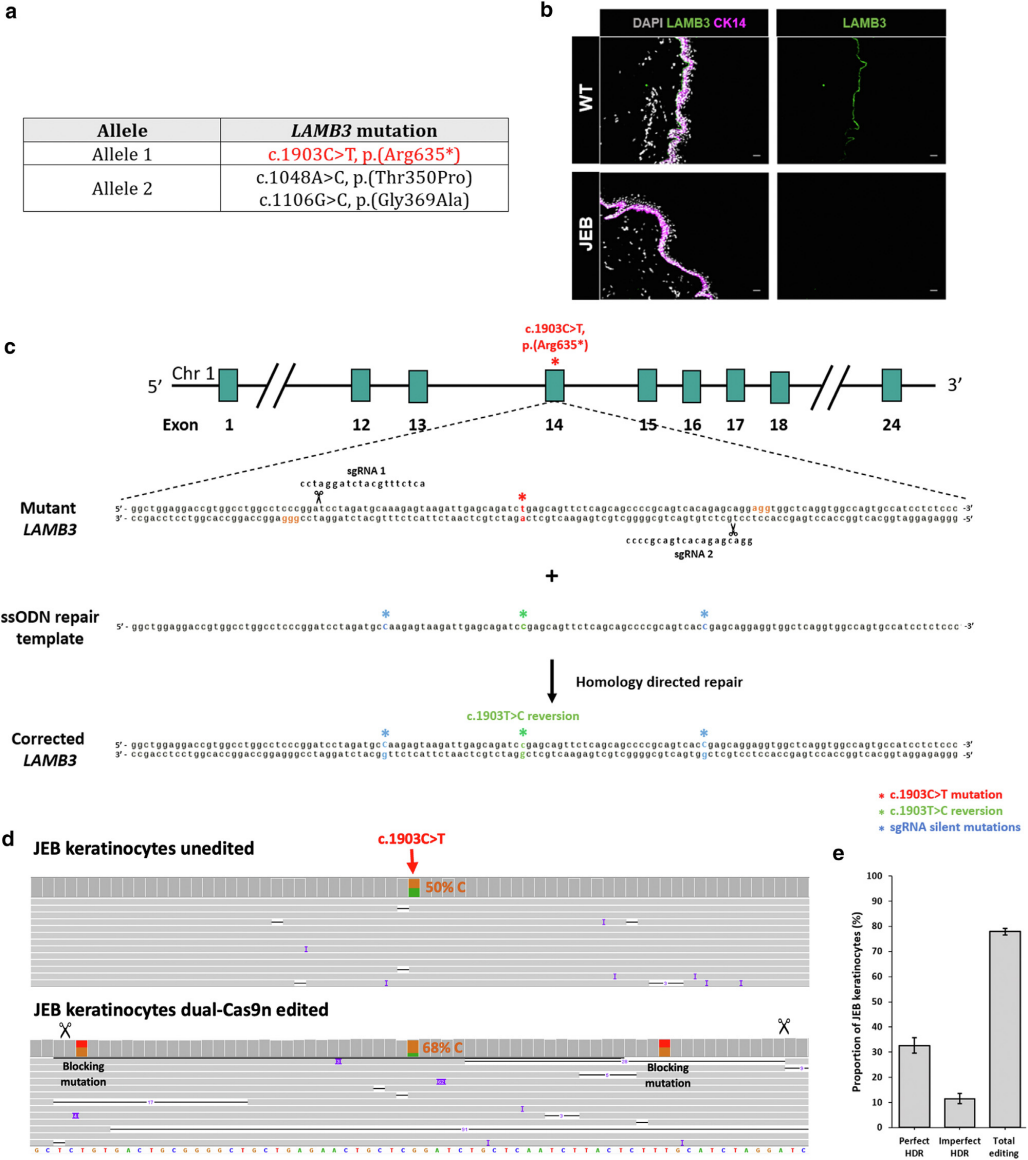
**Dual-Cas9n HDR correction strategy targeting the pathogenic *LAMB3* variant c.1903C>T in primary JEB keratinocytes.** (**a**) The specific pathogenic variants present on both *LAMB3* alleles for the patient with JEB enrolled in this study. (**b**) IHC of skin sections from the patient with JEB compared with that from a healthy donor showing expression of LAMB3 (in green) and the epidermal marker cytokeratin-14 (in magenta). DAPI (in white) was used to stain cell nuclei. Bars = 50 μm. (**c**) Diagrammatic overview of a dual-Cas9n correction strategy targeting the c.1903C>T variant in exon 14 of *LAMB3*. The *LAMB3* allele being targeted and the design of the ssODN repair template used to facilitate HDR correction are shown, with the predicted *LAMB3* allele after correction shown below. The position of the sgRNAs is also shown, with the PAM sites highlighted in orange. (**d**) Raw Nanopore sequencing data aligned to the human reference genome of PCR amplicons generated from genomic DNA from unedited JEB keratinocytes (top) and dual-Cas9n–edited JEB keratinocytes (bottom) viewed in IGV. A histogram along the top displays the read coverage for each nucleotide within the amplicon sequence (reference sequence shown along the bottom). Note that the reference sequence shown along the bottom displays the (−) antisense strand in the opposite direction to subfigure **c**. Each line represents an individual sequencing read. The nucleotide position of the heterozygous c.1903C>T variant is shown and is present at 50% in unedited JEB keratinocytes (50% C: 50% T). The position of the Cas9 nick sites is depicted with scissors, along with the incorporation of the single-blocking SNV in each sgRNA sequence. (**e**) The frequency of perfect HDR, imperfect HDR, and total editing events (combined HDR and INDELs) in dual-Cas9n–edited JEB keratinocytes (n = 3). dual-Cas9n, dual-Cas9 nickase; HDR, homology-directed repair; IGV, Interactive Genomics viewer; IHC, immunohistochemistry; INDEL, insertion and deletion; JEB, junctional epidermolysis bullosa; PAM, protospacer adjacent motif; sgRNA, single-guide RNA; SNV, single nucleotide variant; ssODN, single-stranded oligodeoxynucleotide; WT, wild type.

A donor with JEB was enrolled into our study. Genotyping identified compound heterozygous variants in the *LAMB3* gene (encoding the *β*3 subunit of laminin-332) (Figure 1a). Immunohistochemistry analysis of the *β*3 subunit of laminin-332 in a skin punch biopsy from this donor showed a near null phenotype compared with that from a healthy donor (Figure 1b). Targeting a pathogenic variant in exon 14 (c.1903C>T, p. Arg635∗) (Figure 1c), we employed a dual-Cas9 nickase (dual-Cas9n) repair strategy using the D10A Cas9 nickase variant. Paired sgRNAs flanking the pathogenic variant in exon 14 were designed according to previous research following a ‘protospacer adjacent motif (PAM)-out’ orientation and spaced 66 bp apart (Schubert et al, 2021) (Figure 1c and Supplementary Table S1). For repair templates, we designed short (132 bp) single-stranded oligodeoxynucleotides (ssODNs) symmetrically positioned over the pathogenic variant, with 40 bp homology arms flanking a silent single nucleotide variant (SNV) in each sgRNA sequence (ie, blocking SNVs) to prevent renicking of the genomic DNA (Figure 1c and Supplementary Table S1). We avoided viral vector or plasmid-based delivery methods in favor of electroporation of the sgRNAs and Cas9 nickase as ribonucleoproteins owing to their transient intracellular existence, reduced off-target risk, and robust on-target editing activity (Kim et al, 2014; Seki and Rutz, 2018).

After coelectroporation of dual-Cas9n ribonucleoproteins and ssODNs into primary JEB keratinocytes, we extracted genomic DNA and amplified a ∼700 bp region spanning the pathogenic variant site. The amplicons were genotyped using Nanopore sequencing, and CRISPResso2 (https://crispresso2.pinellolab.org) was used to analyze the sequencing reads (minimum 10,000 analyzed reads) (Supplementary Table S2 shows a list of primer sequences). On-target editing was evident in up to 79.2% of sequencing reads (average = 77.9%, n = 3), with up to 36.1% of total reads corresponding to perfect HDR-mediated correction of the c.1903C>T variant (average = 32.6%, n = 3) (Figure 1d and e and Supplementary Figure S1). On average, an additional 11.6% of sequencing reads corresponded to imperfect HDR whereby partial ssODN integration accompanied with or without INDELs was observed (Figure 1e). Because the goal of our editing strategy was to achieve precise gene repair, we did not characterize these events. However, it is highly likely that a subset of these events would still result in gene correction.

### Highly efficient and precise dual-Cas9n correction of primary JEB keratinocytes using modified ssODNs and a small-molecule HDR enhancer

**Figure 2:**
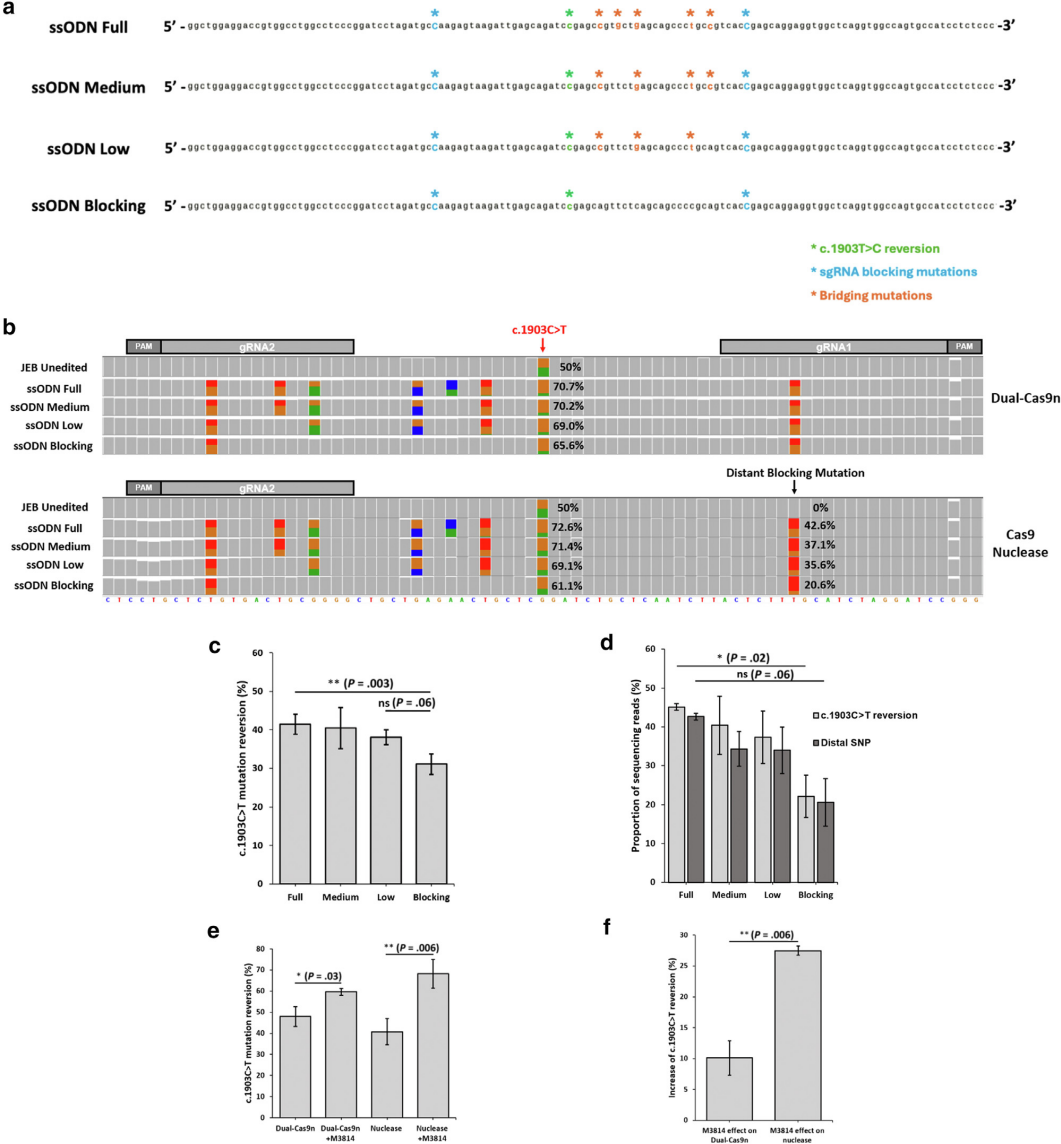
**Highly efficient and precise correction of the pathogenic *LAMB3* variant c.1903C>T in primary JEB keratinocytes using modified ssODN repair templates and a small-molecule HDR enhancer.** (**a**) Design of modified ssODN repair templates with different numbers of silent-bridging SNVs between the sgRNA2 cut site and the pathogenic c.1903C>T variant. (**b**) Raw Nanopore sequencing data aligned to the human reference genome (sequence is shown along the bottom) viewed in IGV showing amplicons generated from genomic DNA from dual-Cas9n–edited (top) or Cas9-sgRNA2–edited (bottom) primary JEB keratinocytes. Note that the reference sequence shown along the bottom displays the (−) antisense strand in the opposite direction to subfigure **a**. A histogram of sequencing read depth is shown for each nucleotide position for the different ssODN templates. The colored bars represent the positions of the silent SNVs within the ssODNs. The locations of the sgRNA sequences are also shown. (**c, d**) The HDR efficiencies achieved using the different ssODNs for (**c**) dual-Cas9n– and (**d**) Cas9-sgRNA2–editing strategies (n = 2). Data represent the mean ± SEM; ∗*P* < .05 and ∗∗*P* < .01. Independent 2-sample *t*-test was performed. (**e, f**) The effect of the HDR enhancer M3814 on HDR efficiencies for (**e**) dual-Cas9n– and Cas9-sgRNA2–editing strategies and (**f**) the different effect size of M3814 on HDR rates between dual-Cas9n– and Cas9-sgRNA2–editing (n = 3). Data represent the mean ± SEM; ∗*P* < .05 and ∗∗*P* < .01. Independent 2-sample *t*-test was performed. dual-Cas9n, dual-Cas9 nickase; HDR, homology-directed repair; IGV, Interactive Genomics viewer; JEB, junctional epidermolysis bullosa; ns, no significance; sgRNA, single-guide RNA; SNV, single nucleotide variant; ssODN, single-stranded oligodeoxynucleotide.

To enhance the rate of perfect HDR correction, we sought to modify the design of our original ssODN repair template (‘ssODN Blocking’). Schubert et al (2021) previously reported that the inclusion of silent ‘bridging’ SNVs in an ssODN template linking the Cas9 cut site (mediated by a gRNA with a PAM-out orientation) to a target integration site increased integration of a small (6 bp) insertion marker. Although this was achieved with Cas9 nuclease, we decided to apply this strategy to our dual-Cas9n strategy given the PAM-out design of both sgRNAs. To this end, we designed 3 additional ssODNs (ssODN full, medium, and low) with different numbers of bridging SNVs between the sgRNA2 nick site and the c.1903C>T variant (Figure 2a and Supplementary Table S1). As a form of positive control, we tested all 4 templates using a high-fidelity Cas9 nuclease in combination with sgRNA2 (Cas9-sgRNA2), while leaving the sgRNA1 blocking SNV (positioned 56 bp away from the sgRNA2 cut site) within the templates to determine the effect of the bridging SNVs on the incorporation of more distant nucleotides (Figure 2a). Overall, ∼80% and ∼90% of total sequencing reads showed evidence of editing for dual-Cas9n and Cas9 nuclease, respectively (Figure 2b and Supplementary Figure S2). In both cases, correction efficiencies appeared to increase with the inclusion of additional bridging SNVs, from an average of 31.1% to 41.4% of sequencing reads for dual-Cas9n (*P* = .003) and 22.1% to 45.1% for Cas9-sgRNA2 (*P* = .02) when comparing ssODN blocking with ssODN full (Figure 2b–d). On average, incorporation of the distant sgRNA1-blocking SNV increased from 20.6% to 42.6% of sequencing reads for Cas9-sgRNA2 editing when comparing the same 2 templates; however, this difference did not reach statistical significance (*P* = .06) (Figure 2b and d). We next investigated a single nicking approach using sgRNA2 in combination with the best performing ssODN (ssODN full) but achieved low HDR rates (1.8% of sequencing reads) (Supplementary Figure S3). On the basis of these results, we proceeded with the dual-Cas9n strategy using ‘ssODN full.’

In an attempt to further increase the efficiency of perfect HDR, we edited a batch of primary JEB keratinocytes from the same donor that had undergone fewer in vitro population doublings. These cells likely contained a smaller population of senescent keratinocytes and a higher proportion of actively dividing cells, which we predicted would be more conducive to HDR-mediated repair. In addition, we tested the small-molecule HDR enhancer M3814, a compound previously demonstrated to enhance HDR-mediated repair by inhibiting the critical NHEJ-repair factor DNA-dependent protein kinase (Riesenberg et al, 2023). In the absence of M3814, up to 54.4% of sequencing reads showed perfect HDR correction using our optimized dual-Cas9n editing strategy (average = 48%, n = 3) and up to 46.1% of reads for Cas9-sgRNA2 (average = 40%, n = 3) (Figure 2e and Supplementary Figure S4). In the presence of M3814, this significantly increased up to 61.2% for dual-Cas9n (*P* = .03, average = 59.7%, n = 3) and 74.5% for Cas9-sgRNA2 (*P* = .006, average = 68.22%, n = 3) (Figure 2e). On average, we found that M3814 facilitated a significantly larger increase in HDR efficiency for Cas9 nuclease (27.5%) than for dual-Cas9n (10.1%) (*P* = .006), most likely reflecting the stronger preference for NHEJ-dependent repair of blunt-end DSBs generated by Cas9 nuclease (Nambiar et al, 2022) (Figure 2f). Assessment of in vitro keratinocyte proliferation after gene editing confirmed no difference in population-doubling rates between the edited cells treated with and without M3814 compared with control keratinocytes (Supplementary Figure S5).

Finally, to screen for potential off-target activity induced by either dual-Cas9n or Cas9 nuclease editing, we performed deep Nanopore sequencing of amplicons spanning the top 8 in silico predicted intragenic off-target sites (4 for each sgRNA) (Supplementary Table S2). Sequencing analysis with CRISPResso2 (10,000 reads analyzed per site minimum) confirmed no detectable off-target editing (Bonferroni corrected *P* = 1) (Supplementary Table S3).

### Cas9-based gene editing targeting the pathogenic *LAMB3* variant c.1903C>T generates large on-target deletions that is significantly increased by small-molecule inhibition of NHEJ

**Figure 3:**
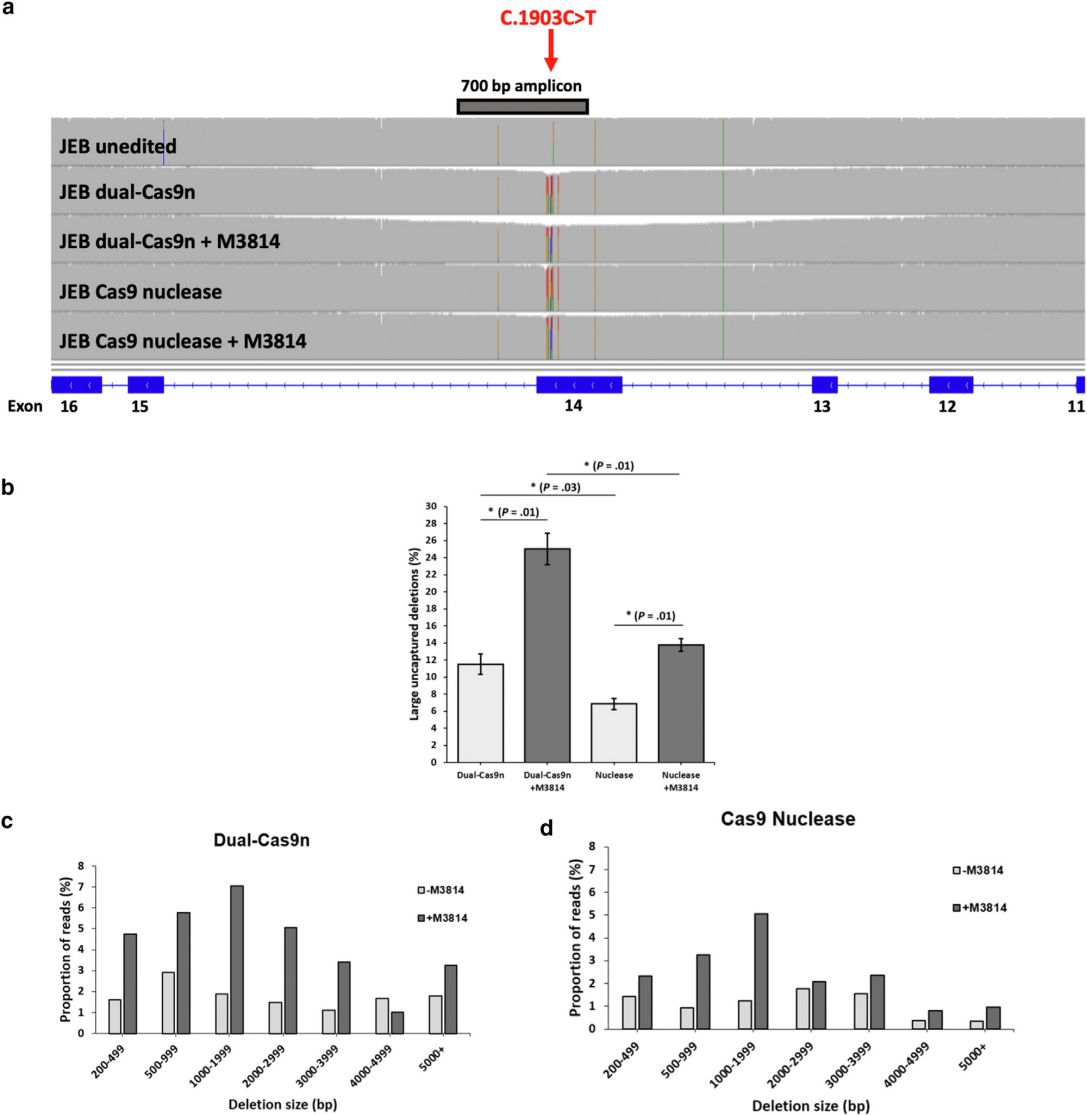
**Cas9-based gene editing generates large on-target deletions that cannot be captured by standard short-range sequencing methods.** (**a**) Raw Nanopore sequencing data aligned to the human reference genome viewed in IGV showing a portion of ∼10.5 kb amplicons generated from genomic DNA after dual-Cas9n and Cas9 nuclease editing in primary JEB keratinocytes. For each sample, a histogram of sequencing read depth across the amplicon is shown. The position of the c.1903C>T variant is shown in red for the unedited JEB sample. The coverage of the 700-bp amplicon used for standard on-target genotyping is depicted with a gray bar. *LAMB3* exons are shown with blue bars along the bottom. Note the reduced coverage spanning the pathogenic variant site in the edited samples compared with that of the unedited control. The colored bars in the histogram represent either the pathogenic variant, the SNVs from the ssODN full template, or low frequency Nanopore sequencing errors. (**b**) The frequency of large on-target deletions generated by dual-Cas9n and Cas9 nuclease editing, which evaded capture by standard on-target genotyping (n = 2). Data represent the mean ± SEM; ∗*P* < .05. Independent 2-sample *t*-test was performed. (**c, d**) Categorization of the deletion sizes generated by (**c**) dual-Cas9n– or (**d**) Cas9 nuclease–editing strategies with and without M3814. dual-Cas9n, dual-Cas9 nickase; IGV, Interactive Genomics viewer; JEB, junctional epidermolysis bullosa; SNV, single nucleotide variant; ssODN, single-stranded oligodeoxynucleotide.

We previously uncovered large, kilobase-sized on-target deletions generated by dual Cas9 nuclease gene editing at the *COL7A1* locus for RDEB (du Rand et al, 2024). Notably, ∼10% of the deletions were too large to be detected by standard genotyping methods. Considering this high frequency, we assessed the on-target deletion profiles resulting from single Cas9 nuclease and dual-Cas9n editing. Nanopore sequencing analysis of long (∼10.5 kb) amplicons symmetrically spanning the c.1903C>T variant revealed large on-target deletions resulting from both modes of editing, which initially evaded capture by sequencing of shorter 700 bp amplicons (Figure 3a and Supplementary Table S2). We observed a significantly higher frequency of these deletions for dual-Cas9n editing (12.7%) than for Cas9 nuclease editing (6.2%) (Figure 3a and b) (*P* = .03). Strikingly, the addition of M3814 resulted in a ∼2-fold increase in the frequency of these events for both editing strategies, amounting to 13.8% for Cas9 nuclease (*P* = .01) and 23.2% for dual-Cas9n (*P* = .01) (Figure 3a and b).

We next categorized the deletion sizes observed from long amplicon sequencing and found unique deletion profiles between Cas9 nuclease and dual-Cas9n editing (Figure 3c and Supplementary Figure S6). Notably, we observed that dual-Cas9n editing generated a higher frequency of large deletions (>5 kb) as a proportion of total deletions than Cas9 nuclease (4.4% for Cas9 nuclease and 14.5% for dual-Cas9n, *P* = .03) (Supplementary Figure S6). The addition of M3814 increased the frequency of deletions across most size categories for both editing strategies, with the largest increase observed for deletions between 1 and 2 kb. In the absence of M3814, approximately 15% of all deletions for both editing strategies were between 1 and 2 kb; in the presence of M3814, this significantly increased to 23% for dual-Cas9n and 30% for Cas9 nuclease (*P* = .01) (Figure 3c and Supplementary Figure S6). For our dual-Cas9n editing strategy, we deemed the high frequency (23%) of large on-target deletions resulting from NHEJ inhibition unacceptable and therefore chose to proceed without the use of the enhancer.

### Precise dual-Cas9n correction of primary JEB keratinocytes rescues expression and secretion of laminin-332 and improves cellular adhesion in vitro

**Figure 4:**
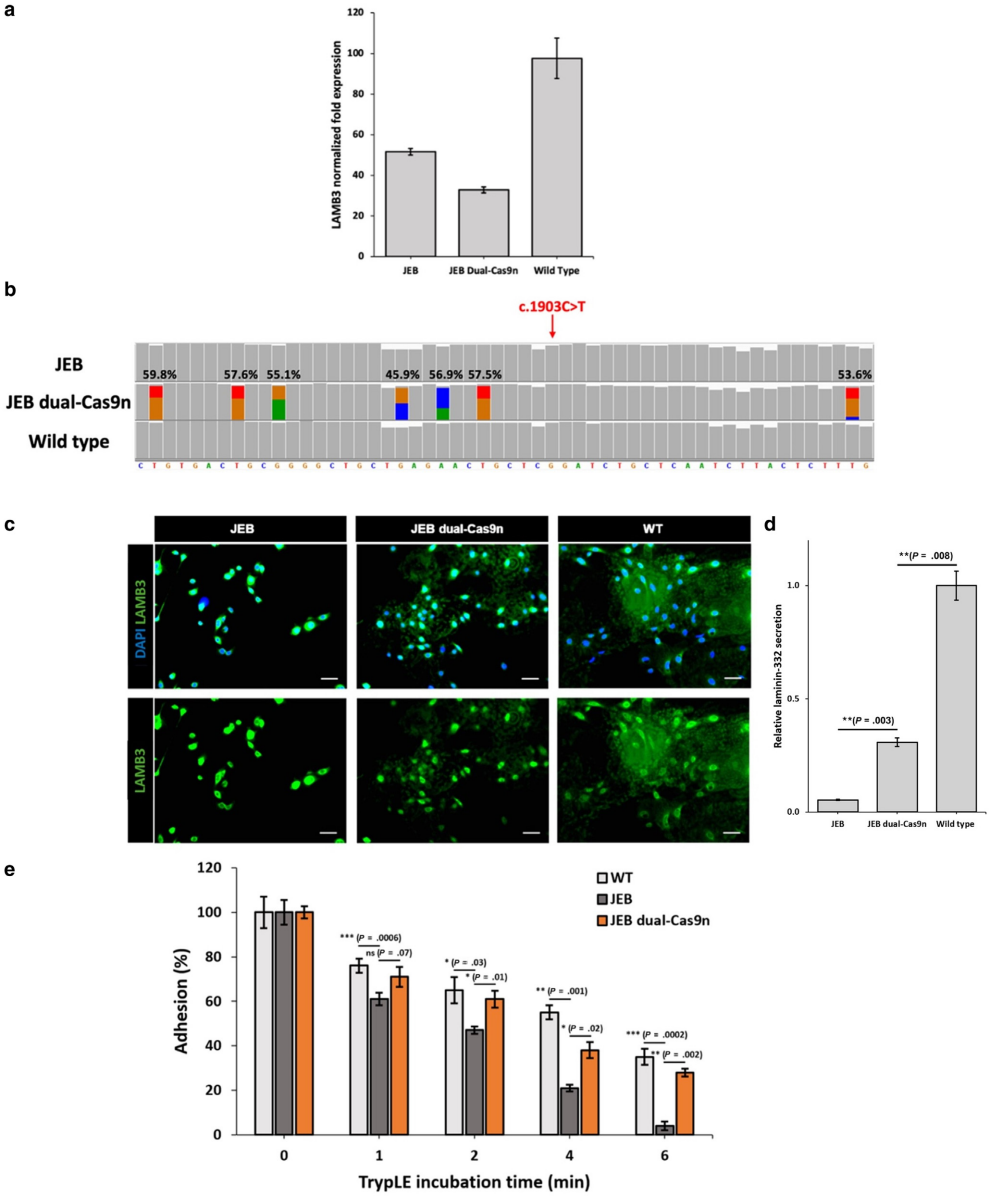
**Precise dual-Cas9n correction of the pathogenic c.1903C>T variant in primary JEB keratinocytes restores laminin-332 secretion and in vitro adhesion.** (**a**) ddPCR quantification of *LAMB3* transcription in unedited and dual-Cas9n–corrected JEB keratinocytes compared with that in 2 WT donors (n = 2). (**b**) Histograms displaying sequencing read depth within exon 14 of *LAMB3* in cDNA amplicons derived from unedited and dual Cas9n–edited JEB keratinocytes compared with that in a WT control. Colored bars represent the silent SNVs from the ssODN repair template incorporated into *LAMB3* mRNA. The frequency of incorporation for each silent SNV is also shown. (**c**) Immunocytochemistry of primary JEB keratinocytes (left panel), dual-Cas9n–treated primary JEB keratinocytes (middle panel), and WT primary keratinocytes (right panel) stained with an anti-LAMB3 antibody (in green). DAPI (in blue) was used to stain cell nuclei (n = 3). Bars = 50 μm. (**d**) Quantification of laminin-332 secretion using ImageJ. Quantification is based on 5 individual images. The data represent the mean ± SEM; ∗*P* < .05 and ∗∗*P* < .01. Independent 2-sample *t*-test was performed. (**e**) In vitro cell adhesion assay displaying the proportion of keratinocytes adhered to cell culture plates after the specified incubation times with TrypLE (n = 2, 5 replicates performed). The data represent the mean ± SEM; ∗*P* < .05, ∗∗*P* < .01, and ∗∗∗*P* < .001. Independent 2-sample *t*-test was performed. ddPCR, droplet digital PCR; dual-Cas9n, dual-Cas9 nickase; JEB, junctional epidermolysis bullosa; SNV, single nucleotide variant; ssODN, single-stranded oligodeoxynucleotide; WT, wild-type.

Next, we assessed *LAMB3* transcription in JEB keratinocytes after dual-Cas9n repair of the c.1903C>T variant. Quantification of *LAMB3* transcription by droplet digital PCR revealed approximately half the level of *LAMB3* mRNA in unedited JEB keratinocytes compared with 2 wild-type donors (Figure 4a). Sequencing of cDNA amplicons derived from *LAMB3* mRNA spanning exons 12–15 confirmed that this discrepancy was due to the null expression of the allele containing the c.1903C>T variant, as evidenced by the complete absence of the variant in the unedited JEB sequence (Figure 4b and Supplementary Figure S7). After dual-Cas9n correction, a reduction in *LAMB3* expression was observed, most likely reflecting INDEL events knocking down the expression of the other allele (Figure 4a). Interestingly, only 40% of cDNA sequencing reads from unedited JEB and wild-type keratinocytes contained exon 14, suggesting a highly frequent alternatively spliced transcript (Supplementary Figure S7). This frequency dropped to 30% after dual-Cas9n editing, potentially a result of large genomic deletions spanning exon 14 (Supplementary Figure S7). Importantly, of the *LAMB3* transcripts containing exon 14, the silent bridging SNVs within the ssODN full repair template showed an average incorporation rate of 55.2%, similar to the 54.4% observed at the genomic level (Figure 4b).

Laminin-332 is a major secreted adhesive ligand for keratinocytes in vitro (Benati et al, 2018). Prior to secretion onto cell culture surfaces, intracellular assembly of the 3 subunits that together form laminin-332—LAMA3, LAMB3, and LAMγ2—is required (Rousselle and Beck, 2013). To investigate LAMB3 expression and secretion in vitro, we performed immunocytochemistry of control and dual-Cas9n–treated primary JEB keratinocytes (Supplementary Figure S8). As expected, LAMB3 expression in healthy keratinocytes was observed both intracellularly and extracellularly coated on the bottom of cell culture vessels (Figure 4c). In contrast, brighter intracellular staining (originating from the other allele) accompanied with a near complete absence of extracellular staining was observed in unedited JEB keratinocytes (Figure 4c). After dual-Cas9n editing, extracellular LAMB3 was observed on the surface of cell culture plates, confirming restoration of LAMB3 expression and secretion from the allele harboring the c.1903C>T variant (Figure 4c). Quantification of secreted laminin-332 demonstrated that dual-Cas9n–corrected JEB keratinocytes secreted approximately 30% of the laminin-332 content compared with wild-type keratinocytes (Figure 4d). This is expected because our strategy can only enable monoallelic LAMB3 expression given the heterozygosity of the targeted pathogenic variant. Therefore, the precise correction observed at the genomic level of up to 54% would be expected to result in protein re-expression from approximately 27% of the total alleles, similar to the observed level (Figure 4d).

Next, to determine whether the restored expression and deposition of laminin-332 observed on cell culture vessel surfaces enhanced keratinocyte attachment, we performed a cell detachment assay. Control and dual-Cas9n–edited JEB keratinocytes were treated with the cell detachment reagent TrypLE for various time points, and the proportion of the remaining cells adhered after these time points was measured. As expected, unedited JEB keratinocytes displayed a poor attachment capacity compared with healthy keratinocytes (Figure 4e). After dual-Cas9n repair, primary JEB keratinocytes showed a significant increase in cell adhesion comparable with healthy keratinocytes (Figure 4e).

### Restoration of laminin-332 expression and accurate deposition to the dermal–epidermal junction in dual-Cas9n–edited engineered SEs

**Figure 5:**
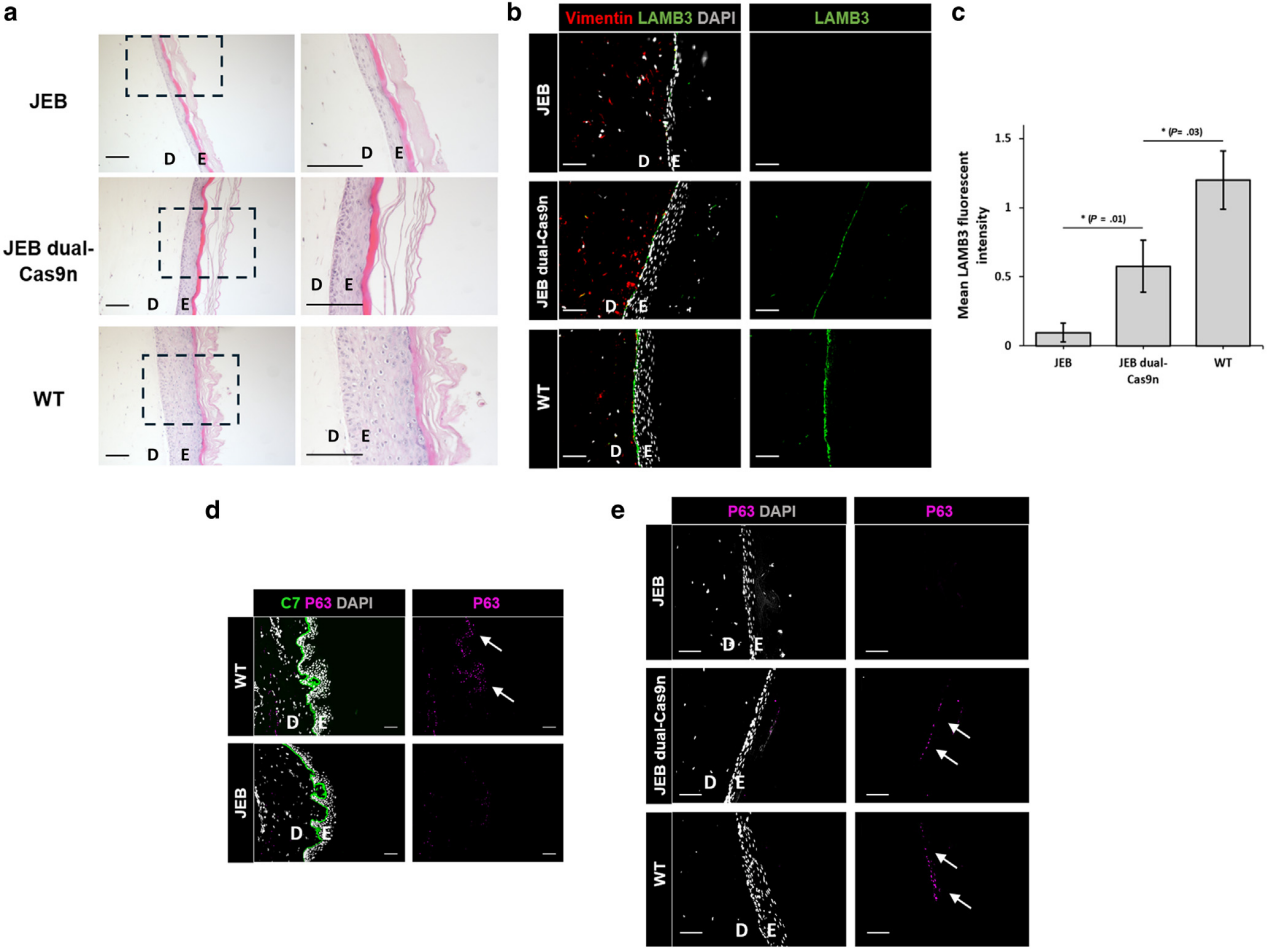
**Accurate laminin-332 deposition to the dermal–epidermal junction in engineered SEs after dual-Cas9n correction of primary JEB keratinocytes.** (**a, b**) Analysis of engineered SEs generated with healthy fibroblasts in combination with either unedited primary JEB keratinocytes (top panel), dual-Cas9n–treated JEB keratinocytes (middle panel), or WT keratinocytes (bottom panel). Structure of engineered SEs is shown by H&E staining in **a**, and immunofluorescent analysis is shown in **b**. The dermal (denoted as D) and epidermal (denoted as E) layers are indicated. Vimentin was used as a dermal marker (in red), and DAPI (in white) was used as a nuclear marker. Bars = 50 μm. (**c**) Quantification of LAMB3 expression in engineered SEs using ImageJ. Four individual images were used for quantification per sample. The data represent the mean ± SEM; ∗*P* < .05. Independent 2-sample *t*-test was performed. (**d**) Immunofluorescence of skin sections from a WT donor and the patient with JEB visualizing the expression of p63α. C7 was used as a basement membrane marker (in green). DAPI (in white) was used as a nuclear marker. Bars = 50 μm. The dermal (denoted as D) and epidermal (denoted as E) layers are indicated. (**e**) Visualisation of p63α expression (in magenta) by immunofluorescence in engineered SEs generated from unedited primary JEB keratinocytes (top panel), dual-Cas9–treated primary JEB keratinocytes (middle panel), and WT primary keratinocytes (bottom panel). The dermal (denoted as D) and epidermal (denoted as E) layers are indicated. C7, type VII collagen; dual-Cas9n, dual-Cas9 nickase; JEB, junctional epidermolysis bullosa; SE, skin equivalent; WT, wild-type.

To assess whether the restored secretion of laminin-332 observed in 2-dimensional culture would be accurately deposited to the dermal–epidermal junction, we engineered 3-dimensional SEs composed of dual-Cas9n–treated JEB keratinocytes (∼50% correction) (Figure 5a). Near complete absence of LAMB3 was observed by immunohistochemistry analysis of SEs containing unedited JEB keratinocytes (Figure 5b). In comparison, SEs containing dual-Cas9n–edited JEB keratinocytes showed accurate laminin-332 deposition linearly along the dermal–epidermal junction (Figure 5b). Quantification of LAMB3 at the dermal–epidermal junction revealed approximately 50% the level of expression in dual-Cas9n–corrected SEs relative to that in wild-type SEs (Figure 5c).

Epidermal thickness within the engineered SEs differed between samples (Figure 5a and b). SEs composed of unedited JEB keratinocytes had a relatively thinner epidermal layer than the thick epidermal layer present in SEs composed of wild-type keratinocytes (Figure 5a and b). Notably, a thickening of the epidermal layer was observed after dual-Cas9n correction (Figure 5a and b). A potential explanation for this finding is the fact that defective laminin-332 signaling in the skin of patients with JEB is known to deplete EpiSCs owing to phosphorylation and sequestration of the key stem cell marker YAP1, precluding its import into the nucleus (De Rosa et al, 2019) (Supplementary Figure S9). Restoration of normal laminin-332 signaling is sufficient to rescue this defect by restoring the nuclear import of YAP1 and subsequent EpiSC function (De Rosa et al, 2019). Therefore, we next assessed the levels of Np63α, a transcription factor stabilized by YAP1 in the nucleus known to underpin the regenerative and proliferative capacity of EpiSCs in human skin (De Rosa et al, 2019). Consistent with previous research (De Rosa et al, 2019; Kueckelhaus et al, 2021), nuclear-localized Np63α expression was evident in donor-derived healthy human skin but nearly undetectable in donor-derived JEB skin (Figure 5d). Similarly, <1% of keratinocytes in unedited JEB SEs showed Np63α expression (Figure 5e). However, on average, 11.5% of keratinocytes in dual-Cas9n JEB SEs showed restored Np63α expression, compared with 19.6% in wild-type SEs (n = 3) (Figure 5e).

## Discussion

The development of an ex vivo gene therapy involving the grafting of genetically modified autologous keratinocyte stem cells represents a promising goal of EB research. To date, the gene therapy landscape for *LAMB3-*defective JEB has been dominated by several successful ex vivo gene replacement approaches reliant on retroviral integration into the genome (du Rand et al, 2022). More recently, the in vivo, topically applied nonintegrating gene therapy Vyjuvek gained Food and Drug Administration approval for treating RDEB after several highly successful clinical trials (Gurevich et al, 2022). Gene editing mediated by designer nucleases confers a potential advantage to gene replacement because it enables permanent and targeted DNA modifications to restore normal protein expression and function. Although this can be efficiently facilitated by end-joining repair, HDR-mediated correction currently represents a more ideal strategy because it can theoretically enable precise repair of any pathogenic variant.

In the past few years, significant improvements in HDR repair have been reported for EB. For example, Petković et al (2022) reported impressive gene correction rates of up to 38% targeting *COL17A1* in primary JEB keratinocytes, demonstrating restoration of accurate protein localization in engineered SEs and reversal of dermal-epidermal blistering. For RDEB, 2 separate groups reported HDR efficiencies of up to 50% (Bonafont et al, 2021) and 58% (Berthault et al, 2024) in primary RDEB skin cells. Although promising, gene correction efficiencies in both instances were based on the assessment of only a limited number of edited clones. In addition, the majority of HDR events reported by Berthault et al (2024) were accompanied with INDELs localized within intron 79, precluding precise gene correction that represents the ultimate goal of gene editing for EB.

Off-target genotoxicity mediated by Cas9 nuclease remains a safety concern and has been reported on multiple occasions for EB (Benati et al, 2018; Bischof et al, 2022; Klermund et al, 2024; Kocher et al, 2021, 2019; Webber et al, 2016). To improve the safety of Cas9-based gene editing, several groups have increasingly adopted dual-Cas9n approaches (Klermund et al, 2024; Kocher et al, 2021, 2019, 2017; Webber et al, 2016). In some of these studies, a substantially high level of unintended Cas9 nuclease editing (including high levels of chromosomal translocations) was reported. Notably, near complete ablation of this toxicity was achieved when using the same sgRNAs in combination with Cas9 nickases (Klermund et al, 2024; Kocher et al, 2021; Webber et al, 2016).

Although dual-Cas9n approaches carry a significantly lower risk of off-target mutagenesis, gene correction rates have fallen short of Cas9 nuclease–targeting strategies, reaching up to 18.3% and 21% targeting *COL17A1* (Petković et al, 2022) or *COL7A1* (Kocher et al, 2021)*,* respectively. Furthermore, it is important to highlight that many EB gene editing studies have focused on the correction of homozygous variants (Berthault et al, 2024; Bonafont et al, 2021; Klermund et al, 2024; Kocher et al, 2021; Webber et al, 2016). Targeting heterozygous variants will likely require further enhancements in gene correction efficiencies because only monoallelic correction can be achieved. In this study, we established a dual-Cas9n strategy targeting a heterozygous *LAMB3* variant, using electroporation to deliver the ribonucleoproteins and ssODNs to ensure robust on-target editing while reducing off-target activity (Kim et al, 2014; Seki and Rutz, 2018). Implementation in primary JEB keratinocytes enabled perfect gene repair evident in up to 54% of 700 bp Nanopore sequencing reads. Using a high-fidelity Cas9 nuclease, we also achieved perfect gene correction efficiencies of up to 74% of reads using a small-molecule HDR enhancer. Preliminary off-target assessment of the top 4 intragenic in silico predicted off-target sites per gRNA did not show any detectable Cas9 cleavage. However, we acknowledge that more rigorous analyses would be necessary before clinical translation. This could include experimental techniques such as GUIDE-seq (genome-wide, unbiased identification of double-stranded breaks enabled by sequencing) capable of identifying bona fide sites of nuclease induced DSBs (Tsai et al, 2015) and CAST-seq to search for large chromosomal aberrations (Klermund et al, 2024).

The inclusion of silent blocking SNVs within the PAM or gRNA site of ssODN repair templates can significantly increase HDR efficiencies by preventing recutting of genomic DNA (Schubert et al, 2021). Previous research has shown that the incorporation of more distal edits can be further increased through the incorporation of additional silent ‘bridging’ SNVs linking the Cas9 cleavage site to the HDR edit site (Paix et al, 2017; Schubert et al, 2021). Schubert et al (2021) demonstrated that this strategy was most effective when using a gRNA with a PAM-out orientation relative to the HDR edit site and an ssODN complementary to the nontarget strand (ie, the PAM-containing strand). To meet these criteria, we positioned various numbers of bridging SNVs adjacent to gRNA2 and observed a significant increase in the reversion of the c.1903C>T variant with the inclusion of additional bridging SNVs. Importantly, we showed that these simple template design modifications can also be effective with dual-Cas9n HDR strategies and should be considered for future editing strategies. It is important to highlight that because HDR is a highly complex process, optimization will be necessary for each locus to determine optimal ssODN design parameters, including the placement and number of bridging SNVs.

Next-generation sequencing of short amplicons is currently the gold standard for characterizing on-target Cas9-mediated editing events. However, our group and others have previously demonstrated that a large frequency of on-target deletions evade capture by these analyses (du Rand et al, 2024; Hunt et al, 2023; Klermund et al, 2024; Owens et al, 2019). In this study, we found that the frequency of these events was ∼2-fold higher when using dual-Cas9n than when using Cas9 nuclease. Similarly, Klermund et al (2024) reported a ∼2-fold increase in large (>200 bp) on-target deletions of up to 50% at the *COL7A1* locus resulting from dual-Cas9n editing, compared with 12% and 32% when using the sgRNAs individually with Cas9 nuclease. Although the magnitude of these events is locus dependent (Owens et al, 2019), they are proving to be routine outcomes of DSB-mediated gene editing and need to be considered to ensure accurate data representation, especially when conducting gene editing with dual-Cas9n.

Analysis of dual-Cas9n–corrected JEB keratinocytes showed a restoration of laminin-332 expression and secretion in vitro as well as increased adhesion capabilities. Efficient correction efficiencies at the genomic level of up to 54% corresponded to approximately 30% LAMB3 secretion relative to wild-type keratinocytes. Given the heterozygosity of the targeted variant, this was expected because only up to 54% of the alleles harboring the pathogenic variant could theoretically re-express LAMB3 protein. In comparison with the levels of restored LAMB3 secretion in vitro, restoration of accurate laminin-332 deposition to the dermal–epidermal junction in engineered SEs was observed at approximately 50% the level of wild-type SEs. One possible explanation for this difference is the selective growth advantage of laminin-332–positive keratinocytes (De Rosa et al, 2019). Owing to restored laminin-332 expression and deposition in SEs after dual-Cas9n correction, this growth advantage could theoretically allow the successfully corrected JEB keratinocytes to outcompete the unedited cells during the 2-week cell culture period owing to restored adhesion and stem cell renewal capacity.

Ultimately, the success of ex vivo gene editing strategies for EB will depend on the transplantation of a sufficient population of EpiSCs. This has been a challenge for dual-Cas9n approaches owing to the rates of gene correction previously achieved. In this study, we reported significantly improved HDR repair rates and showed that corrected SEs had restored nuclear Np63α, a transcription factor underpinning the proliferative and regenerative potential of EpiSCs (De Rosa et al, 2019), within a subset of basal keratinocytes. These preliminary data suggest a rescue of the EpiSC defects characteristic of laminin-332–defective JEB keratinocytes; however, future in vivo experiments will be useful to assess the long-term efficacy of this strategy.

Taken together, we established a retroviral-free, dual-Cas9n gene editing strategy targeting *LAMB3*, with the potential to treat a large group of patients with JEB harboring the targeted hotspot variant (c.1903C>T) (Kiritsi et al, 2013). These data represent a significant improvement in the efficacy of precise HDR repair for EB and potentially open the door for therapeutic gene editing facilitated by Cas9 nickases. Given the simplicity and cost effectiveness of this protocol, it has the potential to be tailored to address other pathogenic EB variants.

## Materials and Methods

### Cell culture and transfection of primary skin cells

Primary keratinocytes were isolated from skin biopsies as previously described (Feisst et al, 2023) from a fully consenting donor with JEB under the ethics approval number 19/STH/47 as provided by the Southern Health and Disability Ethics Committee. Primary dermal fibroblasts and human epidermal keratinocytes, adult were commercially sourced from the ATCC.

Primary dermal fibroblasts were cultured in DF10 medium as previously described (du Rand et al, 2024). Primary keratinocytes were cultured in Kelch’s medium as previously described (Feisst et al, 2023) with the following modifications: DMEM without calcium chloride and glutamine (Gibco) and the addition of GlutaMAX Supplement (Gibco), cultured on a layer of irradiated 3T3-J2 murine embryonic fibroblasts (Kerafast). Electroporation of primary keratinocytes was performed using the P3 nucleofection kit (Lonza Bioscience) and the Amaxa 4D Nucleofector (Lonza Bioscience) under pulse setting CM-137. A total of 2 × 10^5^ primary keratinocytes per sample were electroporated with sgRNA (Integrated DNA Technologies) and either Cas9 nuclease V3 (Integrated DNA Technologies) or Cas9 D10A nickase V3 (Integrated DNA Technologies) complexed as ribonucleoproteins (75 pmol sgRNA, 15.75 pmol Cas9 enzyme, 4.76:1 molar ratio) and 50 pmol of ssODNs (Integrated DNA Technologies) (Supplementary Tables S1 and S2 provide details for gRNA and ssODN sequences). The DNA-dependent protein kinase inhibitor M3814 (MedChemExpress) was added to the cell culture medium of selected samples immediately after electroporation at a final concentration of 1 μM. A full media change was performed for 24 hours, with fresh M3814 (1 μM) added for a further 48 hours.

### Genomic DNA extraction and PCR

Genomic DNA was extracted 3 days after electroporation using the Monarch DNA Purification Kit (New England Biolabs) for on- and off-target genotyping and amplified using primer sets tagged with adaptors required for Nanopore library preparation (Supplementary Table S2 provides a list of primers) using a High-Fidelity Phusion Hot Start II DNA Polymerase (Thermo Fisher Scientific). A LongAmp *Taq* DNA Polymerase (New England Biolabs) was used for amplification of long (∼10 kb) amplicons (Supplementary Table S2). Amplicons were purified with AMPure XP beads (Beckman Coulter) and quantified with the Qubit dsDNA HS Assay Kit (Thermo Fisher Scientific) using a Qubit 2 Fluorometer (Thermo Fisher Scientific).

### Processing of Nanopore sequencing reads

To process Nanopore sequencing reads, fastq sequencing files were aligned to the human reference genome (GRCh38) and filtered for full-length reads using SAMtools (version 1.15) and MiniMap2 (version 2.24). For analysis of cDNA sequencing reads to assess *LAMB3* transcription, fastq files were aligned with minimap2 in splice aware mode using the splice junctions from the primary *LAMB3* transcript (GenBank accession number: NM_000228.3). BAM (binary alignment map) files were generated from all cleaned fastq files and viewed in Interactive Genomics Viewer. Processed fastq files were uploaded into CRISPResso2 (https://github.com/pinellolab/CRISPResso2) to quantify editing events.

For long-range Nanopore sequencing analysis, fastq sequencing files were processed as described earlier using SAMtools and minimap2 and used to generate SAM (sequence alignment map) files. Custom Python scripting was then used to extract unique alleles from the SAM files, including the frequency of each allele. Deletions were quantified by size to assess the deletion profiles resulting from Cas9 nuclease and dual-Cas9n gene editing.

### On- and off-target genotyping with Nanopore sequencing

To prepare Nanopore libraries for sequencing, 200 fmol of amplicon was used with the ligation sequencing V14 PCR barcoding kit (SQK-LSK114 with EXP-PBC001, Oxford Nanopore Technologies). Prepared libraries were run on flongle flow cells (FLO-FLG114, Oxford Nanopore Technologies) and sequenced for 24 hours on a MinION sequencer (Mk1B, Oxford Nanopore Technologies). Basecalling was performed using Guppy basecaller. Cleaned fastq files were uploaded into CRISPResso2 for analysis (https://github.com/pinellolab/CRISPResso2). CRISPRessoCompare was used for off-target analysis to compare control and edited sequences.

### *LAMB3* transcription analysis using RT-PCR and droplet digital PCR

The RNAqueous Total RNA Isolation Kit (Thermo Fisher Scientific) was used to extract total RNA from keratinocytes, and subsequent cDNA synthesis was performed with the SuperScript III First-Strand Synthesis kit (Invitrogen). *LAMB3* transcription was quantified with the QX200 droplet digital PCR system (Bio-Rad Laboratories) using the EvaGreen biding dye. The geometric mean of the 2 housekeeping genes *HPRT-1* and *TBP* was used as a normalization factor to normalize *LAMB3* expression (Supplementary Table S2). QuantaSoft Analysis Pro was used for data analysis.

### In vitro adhesion assay of primary keratinocytes

To assess keratinocyte adhesion, 1 × 10^4^ keratinocytes were seeded into 96-well plates. After 24 hours, the cells were washed with Dulbecco’s PBS (Gibco) twice before incubation with TrypLE (Gibco) for 0, 1, 2, 4, or 6 minutes. The cells were washed 2 more times with Dulbecco’s PBS and subsequently fixed and stained in a single step using a 0.5% crystal violet solution (including 10% methanol) (Sigma-Aldrich) for 10 minutes. After 2 further Dulbecco’s PBS washes, a 1% SDS solution (Sigma-Aldrich) was used to lyse the cells for 1 hour. Absorbance was measured using a spectrophotometer at 590 nm to assess the percentage of adherent cells.

### Immunofluorescence staining of primary keratinocytes and skin sections

To prepare keratinocytes for immunofluorescent staining, 5 × 10^3^ cells were seeded into 8-well chamber slides (Nunc). After 3 days, the cells were fixed with 4% paraformaldehyde (Thermo Fisher Scientific) and then permeabilized with 0.5% Triton X-100 (Sigma-Aldrich). The cells were washed 3 times with Tris Buffered Saline (Sigma-Aldrich) and then blocked for 10 minutes at room temperature with 0.25% casein in Tris Buffered Saline containing 10% human serum (Thermo Fisher Scientific). For primary detection, antibody cocktails diluted in dilution buffer (Tris Buffered Saline with 10% human serum) were applied to the cells and incubated overnight at 4 °C (Supplementary Table S4 provides the list of antibodies). After 3 more Tris Buffered Saline washes, secondary antibody cocktails diluted in dilution buffer were applied to the cells for 30 minutes at room temperature (Supplementary Table S4). DAPI was included at 2.5 μg/ml to stain cell nuclei. Skin sections from engineered SEs or human skin biopsies were stained as described earlier. Imaging was performed using a Nikon Ni-U Upright microscope. LAMB3 expression was quantified using ImageJ.

### Generation and analysis of engineered SEs

Engineered SEs were generated as previously described (Carlson et al, 2008) with a few modifications. In brief, 1 × 10^5^ fibroblasts were seeded into a 3 mg/ml bovine type I collagen gel composed of collagen (Organogenesis, Canton, MA), 10% fetal bovine serum (Moregate), GlutaMAX (Thermo Fisher Scientific), MEM (Thermo Fisher Scientific), and sodium bicarbonate (Sigma-Aldrich). Gels were set within Transwell inserts (Organogenesis) and then covered with DMEM (Gibco) containing 10% fetal bovine serum and left for 1 week at 37 °C to contract. Primary keratinocytes that had been cultured in Kelch’s medium for 1 passage in 5% fetal bovine serum were then resuspended in epidermalization medium 1 (containing DMEM without calcium chloride and glutamine [Gibco], Ham’s F-12 [3:1 DMEM to Ham’s F-12], 40 μM adenine [Sigma-Aldrich], 20 nM triiodothyronine [Sigma-Aldrich], 1 μM hydrocortisone [Sigma-Aldrich], 4 mM L-glutamine [Gibco], 10 μg/ml insulin [Sigma-Aldrich], 2 nM progesterone [Sigma-Aldrich], 0.4 μM SB 772077B, 20 ng/μl keratinocyte GF, and 0.1% fetal bovine serum), and 5 × 10^5^ keratinocytes were seeded onto the SEs and left at 37 °C to adhere for 1 hour. SEs were then submerged with epidermalization medium 1 and left for 3 days at 37 °C. The medium was then replaced with epidermalization medium 2 (the same as epidermalization medium 1 + 1.8 mM calcium chloride [Sigma-Aldrich]). SEs were cultured for 5 days at 37 °C. The medium was replaced every 2 days. The SEs were then raised to an air–liquid interface for 1 week (with medium changes every 2 days) in cornification medium (containing DMEM without calcium chloride and Glutamine [Gibco], Ham’s F-12 [1:1 DMEM to Ham’s F-12], 20 nM triiodothyronine, 40 μM adenine, 1.8 mM calcium chloride, 10 μg/ml insulin, 4 mM L-glutamine, and 2% fetal bovine serum).

The SEs were then cut in half, with one half paraffin embedded and the other embedded and frozen in optimal cutting temperature compound as described previously (Carlson et al, 2008). For the paraffin-embedded tissue blocks, a microtome was used to cut 5-μM sections, which were then stained for H&E using standard protocols. Brightfield imaging was performed using a Leica DMR microscope. For the optimal cutting temperature blocks, a Leica Cryotome (CM1860UV) was used to cut 5-μM sections for immunohistochemistry.

### Statistical analysis

Independent 2-sample *t*-tests were conducted in RStudio (version 2021.09.0) to determine statistically significant differences between control and test samples. ∗*P* < .05, ∗∗*P* < .01, and ∗∗∗*P* < .001 were used to denote the levels of significance. The mean ± SEM was used to represent the data.

## Ethics Statement

This study was approved by the Southern Health and Disability Ethics Committee, with approval number 19/STH/47. Written, informed consent was obtained from all patients and control participants.

## Data Availability Statement

The data that support the findings of this study are available from the corresponding author (h.sheppard@auckland.ac.nz) upon request. No datasets were generated or analyzed during this study.

## ORCIDs

Alex du Rand: http://orcid.org/0000-0002-9481-4149

John Hunt: http://orcid.org/0000-0003-3095-0122

Daniel Verdon: http://orcid.org/0000-0001-6060-0065

Ben Buttle: http://orcid.org/0009-0000-0320-8569

Rod Dunbar: http://orcid.org/0000-0001-9626-2600

Diana Purvis: http://orcid.org/0000-0001-7511-7623

Vaughan Feisst: http://orcid.org/0000-0002-3455-1201

Hilary Sheppard: http://orcid.org/0000-0003-1147-4618

## Conflict of Interest

The authors state no conflict of interest.
