# Supplementary material for: Efficient Dual Cas9 Nickase Correction of a Prevalent Pathogenic LAMB3 Variant for Junctional Epidermolysis Bullosa
Source: JID Innov. 2024 Dec 24;5(3):100343. doi: 10.1016/j.xjidi.2024.100343 (PMC11815943; doi:10.1016/j.xjidi.2024.100343)
Supplement: Supplementary Figures 1-9 and Tables 1-4 [file mmc1.docx]

**SUPPLEMENTARY INFORMATION**

**Figure S1**. **Raw sequencing analysis output generated by CRISPResso2 following dual-Cas9n editing with the ssODN Blocking repair template in primary JEB keratinocytes.** (a) The number of reads in the input fastq files uploaded into CRISPResso2, the number of reads after preprocessing, and the number of reads after alignment to the expected amplicon sequence. Note that the number of reads before and after preprocessing and alignment do not change as the sequencing reads were manually cleaned before CRISPResso2 analysis. (b) Analysis of editing event frequency showing the proportion of reads aligned either to the *LAMB3* allele containing the c.1903C>T variant, the wild type *LAMB3* allele, or the HDR allele (following incorporation of the ssODN Blocking repair template). ‘NHEJ’ sequence reads align most closely to the wild type allele. Imperfect HDR represents reads with partial incorporation of the ssODN template with or without INDELs. (c and d) Overview of the most frequent INDEL products surrounding each gRNA cut site for the wild type allele (c) and the allele containing the pathogenic variant (d) resulting from dual-Cas9n editing. Note that HDR events are not shown here. The cleavage site for each gRNA is depicted with a dotted line. Deletions, insertions and substitutions are also shown. The asterisk (*) denotes the unmodified sequence for either the sequence containing the pathogenic variant or wild type sequence. The frequency (%) of each sequence represents the proportion of that sequence out of the total sequences on either the wild type (c) or allele containing the pathogenic variant (d), specifically around the specified gRNA site.


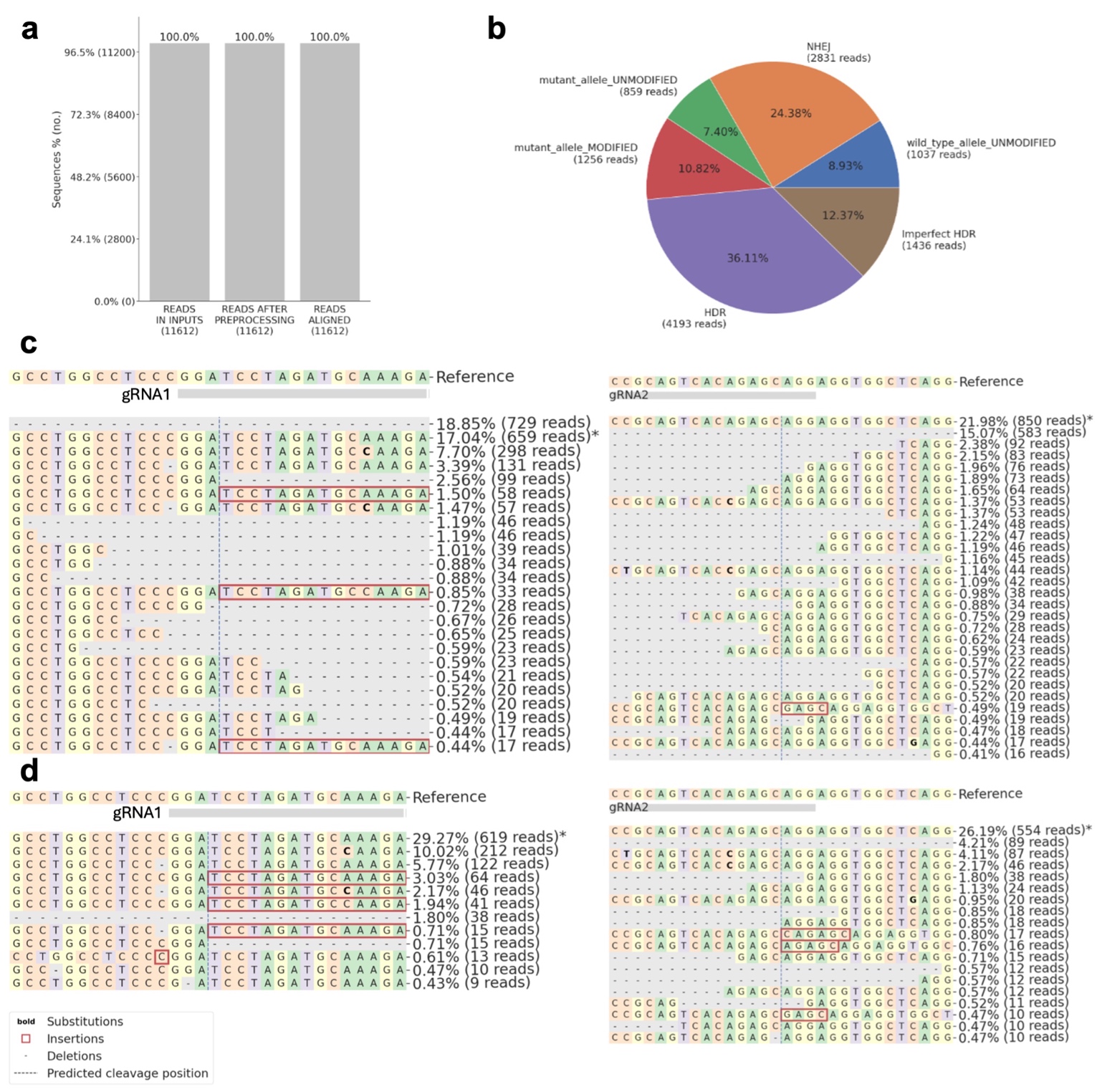


**Figure S2**. **Cas9-mediated editing efficiencies with the different ssODN templates for dual-Cas9n and sgRNA2-Cas9 nuclease editing strategies.** Editing frequency represents the proportion of total sequencing reads with evidence of editing (combined INDELs and HDR) as analysed with CRISPResso2. The data represent the mean ± SEM (n=2).


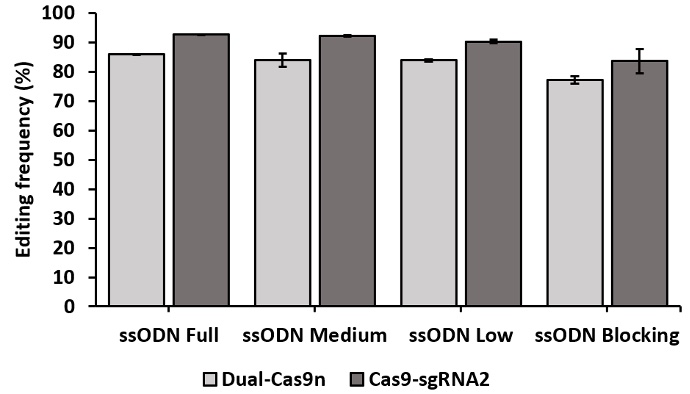


**Figure S3**. **Raw sequencing analysis output generated by CRISPResso2 following sgRNA2-Cas9n single nicking editing with the ssODN Full repair template in primary JEB keratinocytes.** (a) The number of reads in the input fastq files uploaded into CRISPResso2, the number of reads after preprocessing, and the number of reads after alignment to the expected amplicon sequence. Note that the number of reads before and after preprocessing and alignment do not change as the sequencing reads were manually cleaned before CRISPResso2 analysis. (b) Analysis of editing event frequency showing the proportion of reads aligned either to the *LAMB3* allele containing the c.1903C>T variant, the wild type *LAMB3* allele, or the HDR allele (following incorporation of the ssODN Blocking repair template). ‘NHEJ’ sequence reads align most closely to the wild type allele. Imperfect HDR represents reads with partial incorporation of the ssODN template with or without INDELs.


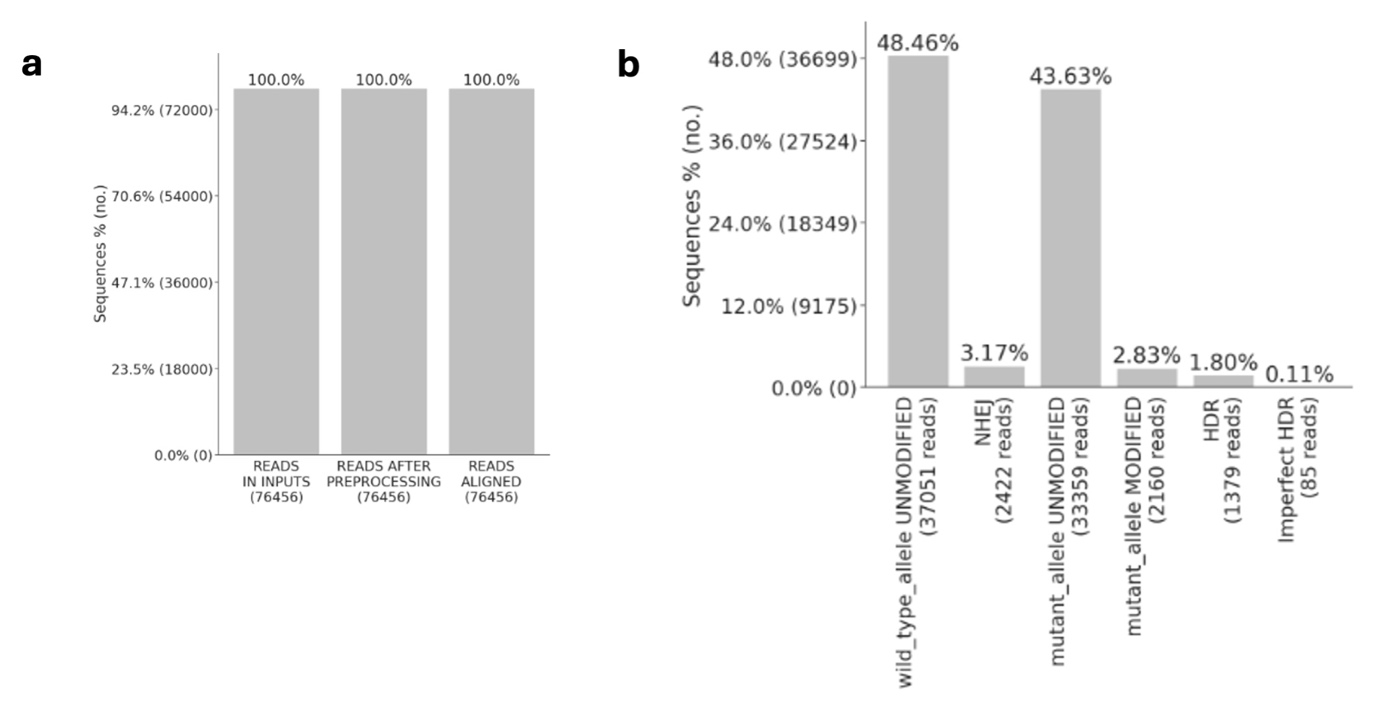


**Figure S4**. **Raw sequencing analysis output generated by CRISPResso2 following optimized dual-Cas9n editing with the ssODN Full repair template in primary JEB keratinocytes.** (a) The number of reads in the input fastq files uploaded into CRISPResso2, the number of reads after preprocessing, and the number of reads after alignment to the expected amplicon sequence. Note that the number of reads before and after preprocessing and alignment do not change as the sequencing reads were manually cleaned before CRISPResso2 analysis. (b) Analysis of editing event frequency showing the proportion of reads aligned either to the *LAMB3* allele containing the c.1903C>T variant, the wild type *LAMB3* allele, or the HDR allele (following incorporation of the ssODN Blocking repair template). ‘NHEJ’ sequence reads align most closely to the wild type allele. Imperfect HDR represents reads with partial incorporation of the ssODN template with or without INDELs. Overview of the INDEL profile surrounding each gRNA cut site for the wild type allele (c) and the allele containing the pathogenic variant (d) resulting from dual-Cas9n editing. Note that HDR events are not shown here. The cleavage site for each gRNA is depicted with a dotted line. Deletions, insertions and substitutions are also shown. The asterisk (*) denotes the unmodified sequence for either the sequence containing the pathogenic variant or wild type sequence. The frequency (%) of each sequence represents the proportion of that sequence out of the total sequences on either the wild type allele (c) or the allele containing the pathogenic variant (d), specifically around the specified gRNA site.


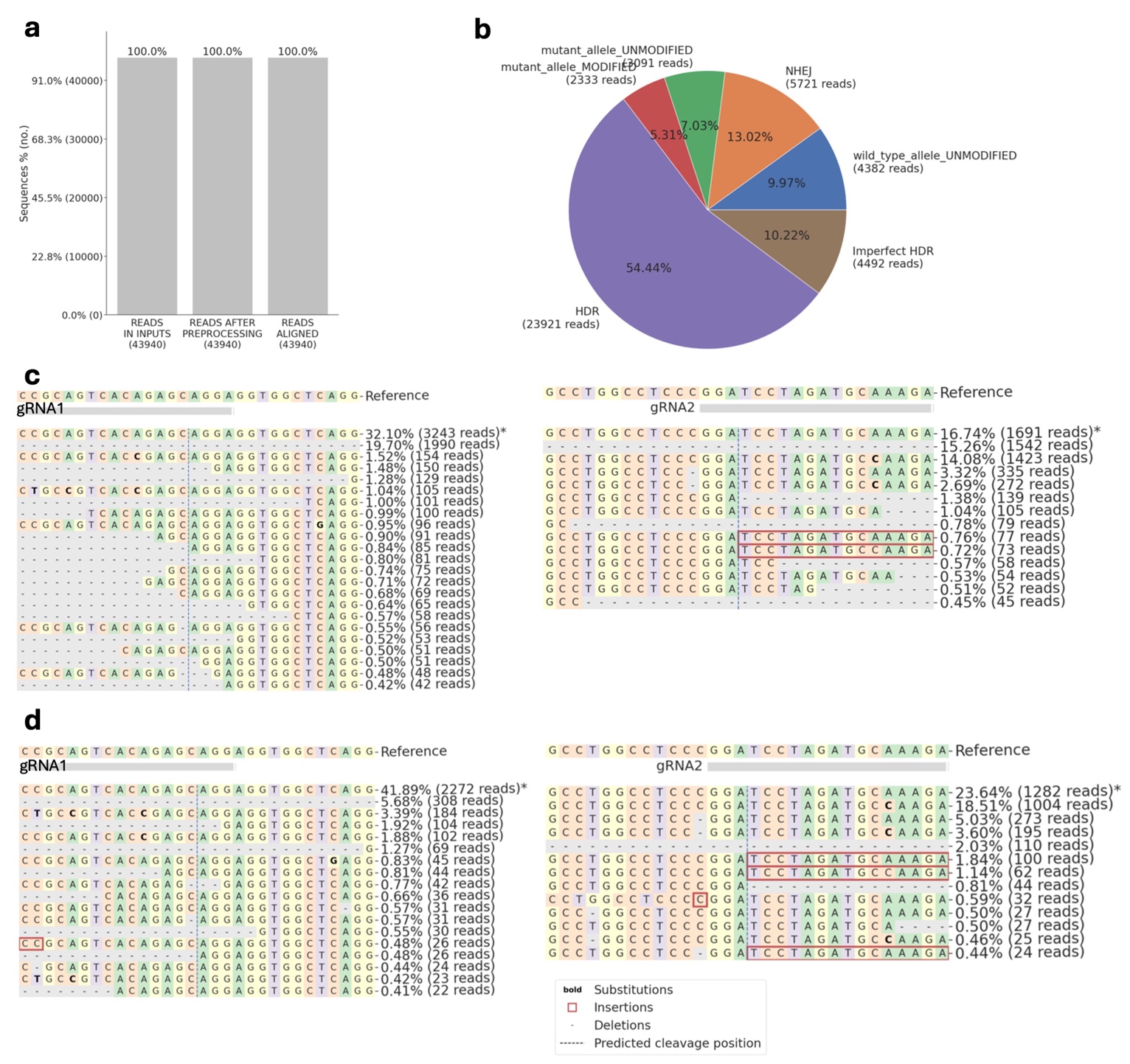


**Figure S5**. **Proliferation analysis of primary keratinocytes**. The graph shows the number of population doublings based on cell counts following dual-Cas9n gene editing with and without M3814, compared to an unedited and wild type control. Day 0 represents the day of dual-Cas9n editing. Data are representative of two individual experiments.


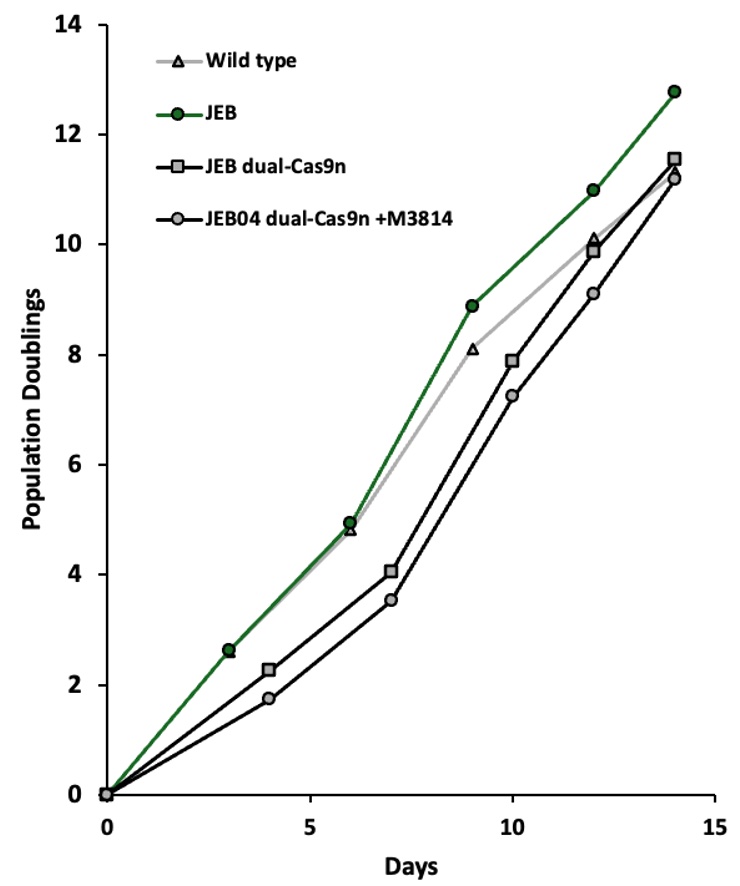


**Figure S6**. **Analysis of on-target deletions >200bp generated by dual-Cas9n and Cas9 nuclease gene editing at the *LAMB3* locus.** (a) The frequency of deletion sizes for Cas9 nuclease and dual-Cas9n editing with and without the small molecule inhibitor M3814 (n=2). (b) Comparison of the frequency of large (>200bp) deletions >5kb generated by dual-Cas9n and Cas9 nuclease (n=2). The data represent the mean ± SEM; p-value < 0.05 (*), p-value < 0.01(**). Independent two-sample t-test performed. (c) The effect of M3814 on the frequency of large (>200bp) deletions between 1-2 kb following gene editing with Cas9 nuclease or dual-Cas9n (n=2). The data represent the mean ± SEM; p-value < 0.05 (*), p-value < 0.01(**). Independent two-sample t-test performed.


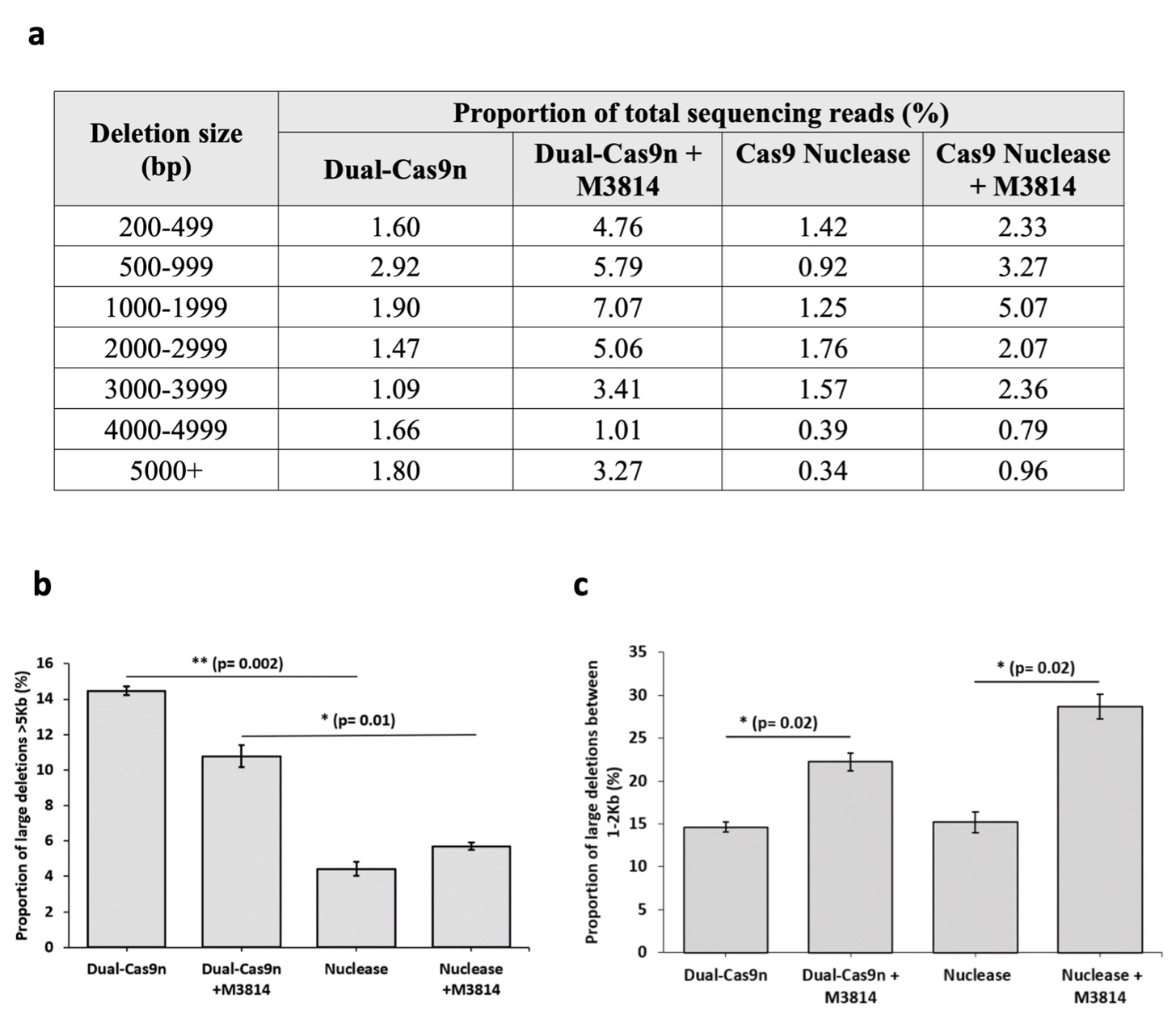


**Figure S7**. **Analysis of cDNA amplicons derived from dual-Cas9n corrected primary JEB keratinocytes.** (a) Raw sequencing data of cDNA amplicons spanning exons 12-15 of *LAMB3* mRNA aligned to the reference human genome and viewed in IGV. For each sample, a histogram of sequencing read depth across the exons (shown at the bottom) is depicted. The percentage of reads containing exon 13 and 14 is shown. The pathogenic variant c.1903C>T is highlighted in red. There is an additional SNV (coloured bar in exon 14) in all samples which represents a Nanopore sequencing error as a result of the surrounding homopolymer sequence. (b) The same cDNA amplicons from (a) run on an agarose gel, showing amplicons containing all four exons (Full transcript), amplicons lacking exon 13 (exon 13 skipped transcript) and amplicons lacking exon 14 (exon 14 skipped transcript). Note that the intensity of the PCR bands do not match the frequency of the transcripts detected by Nanopore sequencing. This is most likely because shorter reads are preferentially sequenced with Nanopore sequencing.


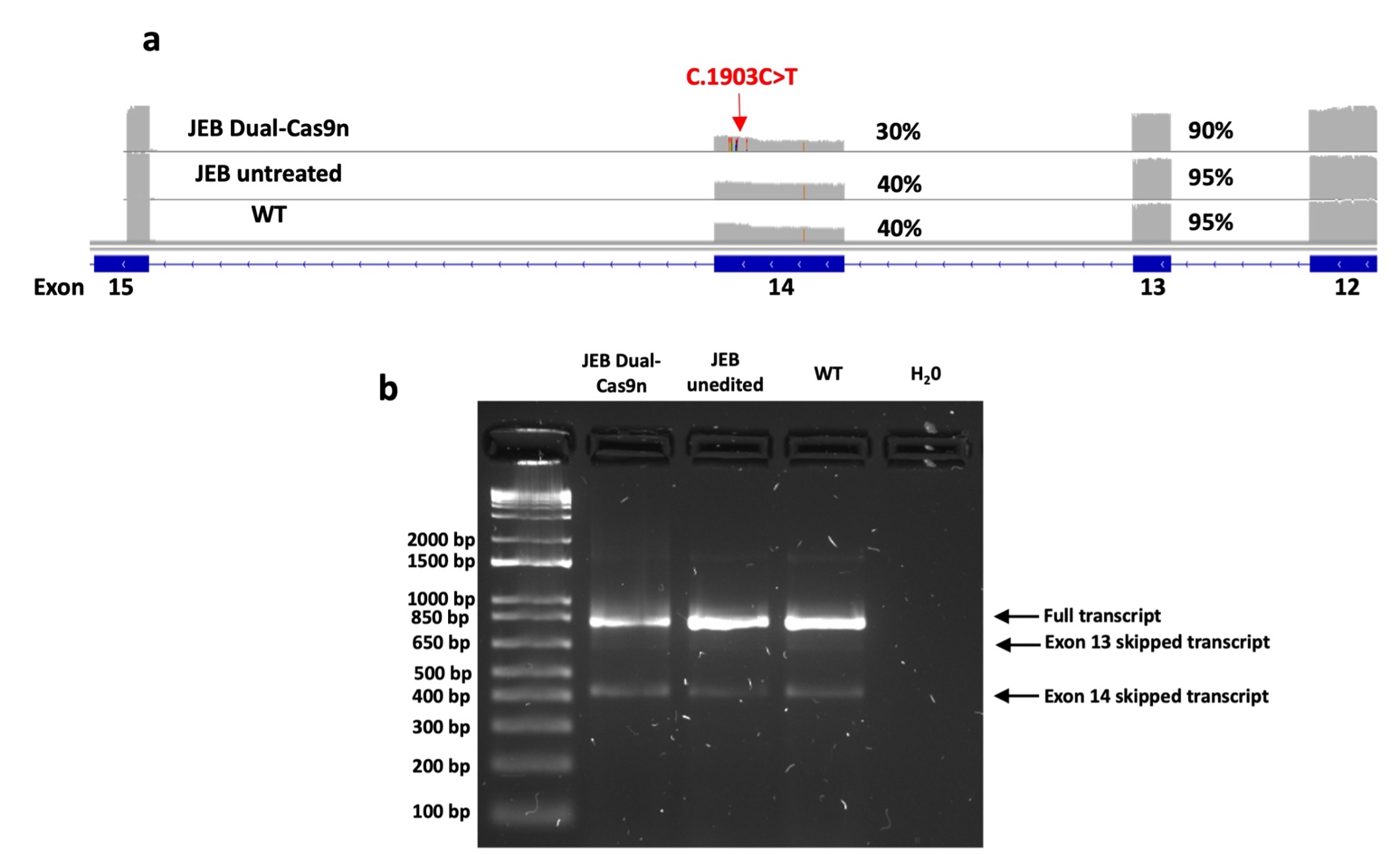


**Figure S8**. **Cytokeratin 14 (CK14) staining of the primary JEB and wild type (WT) keratinocytes used in this study to confirm a pure population of keratinocytes**. CK14 is shown in green. DAPI was used to stain cell nuclei (in blue). Scale bars = 50 µm.


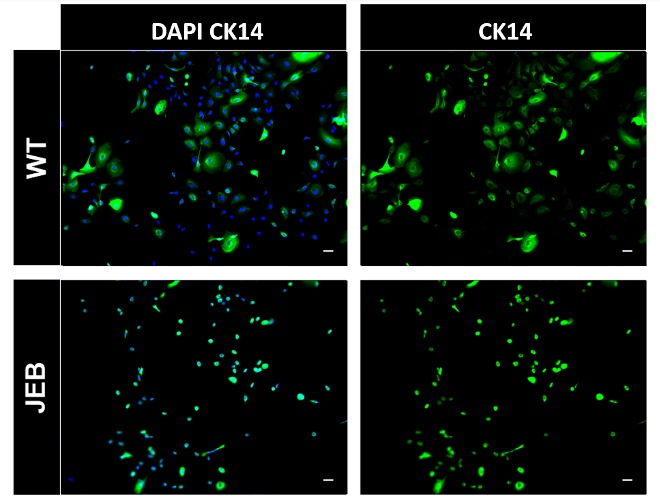


**Figure S9**. **Staining of YAP1 in skin sections from the JEB donor enrolled in this study compared to a wild type (WT) donor**. Nuclear-specific staining is observed in WT skin compared to both nuclear and cytoplasmic staining in JEB skin due to defective laminin-332. Type VII collagen (C7) was used as a basement membrane marker (in green) and DAPI as a nuclear marker (in blue). Scale bars = 50 µm.


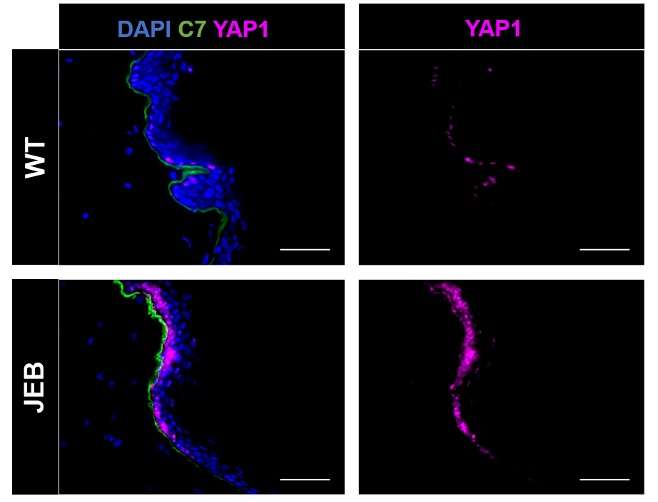


| **gRNA/ssODN** | **Sequence (5’-3’)** |
| --- | --- |
| **LAMB3 gRNA1** | ACTCTTTGCATCTAGGATCC-**GGG** |
| **LAMB3 gRNA2** | CCCCGCAGTCACAGAGCAGG-**AGG** |
| **ssODN Blocking** | GGCTGGAGGACCGTGGCCTGGCCTCCCGGATCCTAGATGC**C**AAGAGTAAGATTGAGCAGATC**C**GAGCAGTTCTCAGCAGCCCCGCAGTCAC**C**GAGCAGGAGGTGGCTCAGGTGGCCAGTGCCATCCTCTCCC |
| **ssODN Low** | GGCTGGAGGACCGTGGCCTGGCCTCCCGGATCCTAGATGC**C**AAGAGTAAGATTGAGCAGATC**C**GAGC**C**GTTCT**G**AGCAGCCC**T**GCAGTCAC**C**GAGCAGGAGGTGGCTCAGGTGGCCAGTGCCATCCTCTCCC |
| **ssODN Medium** | GGCTGGAGGACCGTGGCCTGGCCTCCCGGATCCTAGATGC**C**AAGAGTAAGATTGAGCAGATC**C**GAGC**C**GTTCT**G**AGCAGCCC**T**GC**C**GTCAC**C**GAGCAGGAGGTGGCTCAGGTGGCCAGTGCCATCCTCTCCC |
| **ssODN Full** | GGCTGGAGGACCGTGGCCTGGCCTCCCGGATCCTAGATGC**C**AAGAGTAAGATTGAGCAGATC**C**GAGC**C**GT**G**CT**G**AGCAGCCC**T**GC**C**GTCAC**C**GAGCAGGAGGTGGCTCAGGTGGCCAGTGCCATCCTCTCCC |

**Table S1**. **List of gRNA and ssODN sequences**. The target sequence for each gRNA is shown with the PAM sites in bold. The four ssODN templates used to correct the c.1903C>T variant are shown. All templates encode the correction of the c.1903C>T variant (underlined in red) and contain a silent blocking SNV in each of the gRNA sequences (underlined in green). In addition, the templates contain different numbers of silent bridging SNVs linking the gRNA2 cleavage site and the c.1903C>T variant: ssODN Blocking contains no silent bridging SNVs; ssODN Low contains three silent bridging SNVs; ssODN Medium contains four silent bridging SNVs; and ssODN Full contains five silent bridging SNVs.

| **Primer Name** | **Sequence (5’-3’)** |
| --- | --- |
| **LAMB3 Exon 14 On-Target** | **FWD**: TTTCTGTTGGTGCTGATATTGCGCGAGGCTACTGTAATCGCT  **REV**: ACTTGCCTGTCGCTCTATCTTCCGGGACCAACTTCCATTCCA |
| **LAMB3 Exon 14 On-Target Long Amplicon** | **FWD**: TTTCTGTTGGTGCTGATATTGCCTTTGACCCCGCTGTGTTTG  **REV**: ACTTGCCTGTCGCTCTATCTTCCTCCTCCCCATCTCTCCCAA |
| **gRNA1 OT1** | **FWD**: TTTCTGTTGGTGCTGATATTGCACAGGAGAGCAGAGAAGTGC  **REV**: ACTTGCCTGTCGCTCTATCTTCACAAAGAGACCCCAACCGTC |
| **gRNA1 OT2** | **FWD**: TTTCTGTTGGTGCTGATATTGCGGGAGTTTGACCTCTGCCTC  **REV**: ACTTGCCTGTCGCTCTATCTTCAATAACACCTGCGCCTCCTT |
| **gRNA1 OT3** | **FWD**: TTTCTGTTGGTGCTGATATTGCTTTGAACCTGTCCTGGCCTC  **REV**: ACTTGCCTGTCGCTCTATCTTCAAGCCGACAACCTCTTTCCC |
| **gRNA1 OT4** | **FWD**: TTTCTGTTGGTGCTGATATTGCCTACTGTAGAGGCCGCCTTG  **REV**: ACTTGCCTGTCGCTCTATCTTCCTGTCTGCTTCCCCTGCTTG |
| **gRNA2 OT1** | **FWD**: TTTCTGTTGGTGCTGATATTGCACAGCTTTCAGGAGCCATCC  **REV**: ACTTGCCTGTCGCTCTATCTTCTCACTCCTCCTTCTCCTGGG |
| **gRNA2 OT2** | **FWD**: TTTCTGTTGGTGCTGATATTGCTGTCATGGTTGCCGGTACTC  **REV**: ACTTGCCTGTCGCTCTATCTTCGCGGTGCTTGTGCTACATTC |
| **gRNA2 OT3** | **FWD**: TTTCTGTTGGTGCTGATATTGCGCCTCTCCACATTCTTGGCT  **REV**: ACTTGCCTGTCGCTCTATCTTCCCCCACTTGAACTCACTCCC |
| **gRNA2 OT4** | **FWD**: TTTCTGTTGGTGCTGATATTGCGCCAGACTAGACCAGAGTGTC  **REV**: ACTTGCCTGTCGCTCTATCTTCCACAAAGACGCGCTATGCC |
| ***LAMB3* cDNA Exon 12-15** | **FWD**:TTTCTGTTGGTGCTGATATTGCTGTGACTGCAACATCCTGGG  **REV**:ACTTGCCTGTCGCTCTATCTTCCTCTCGGAAGGGACAACGTC |
| ***LAMB3* ddPCR Exon 23** | **FWD**: ATGGAGTTGGAGCTGCTG  **REV**: ATGTGGTCACGGATCTGC |
| **TBP ddPCR Housekeeper** | **FWD**: GAGTTCTGGGATTGTACCGC  **REV**: CACGAAGTGCAATGGTCTTT |
| **HPRT-1 ddPCR Housekeeper** | **FWD**: GCTGAGGATTTGGAAAGGGT  **REV**: CCTTCATCACATCTCGAGCAA |

**Table S2**. **List of all primer sequences**. Tagged adaptor sequences required for Nanopore library preparation are highlighted in red. OT - off-target; cDNA - complementary DNA; ddPCR – digital droplet PCR

| **Off-target site** | **Sequence (5’-3’)** | **Genomic location (Chr, coordinates, gene)** |
| --- | --- | --- |
| **sgRNA1 OT1** | ACTCTTTGCATC**C**AGGA**G**CC | Chr5, 51,377,229, *CTD-2314G24.2* |
| **sgRNA1 OT2** | **C**CTCTTTGC**C**TCT**G**GGATCC | Chr16, 58,030,415, *MMP15* |
| **sgRNA1 OT3** | **C**CTCTT**A**GCATCT**G**GGATCC | Chr7, 29,207,463, *EMID1* |
| **sgRNA1 OT4** | **G**C**G**CTTTG**A**ATCTAGGATCC | Chr3, 113,230,170, *BOC* |
| **sgRNA2 OT1** | **G**CCCG**A**AGTCACAGAGCAGG | Chr17, 19,717,020, *SLC47A2* |
| **sgRNA2 OT2** | CCC**T**GCAG**A**CACAG**G**GCAGG | Chr2, 2,098,633, *MYTIL* |
| **sgRNA2 OT3** | CCCCG**AG**GTCACAGAGCA**A**G | Chr3, 141,080,959, *SPSB4* |
| **sgRNA2 OT4** | C**T**CC**C**CAGT**G**ACAGAGCAGG | Chr10, 11,893,613, *PROSER2-AS1* |

**Table S3**. Off-target sites analysed for gRNA1 and gRNA2 targeting *LAMB3*. The top four intragenic sites per gRNA were chosen for analysis. The sequence of the off-target site (excluding the PAM sequence) is shown, with the nucleotide mismatches to the gRNA highlighted in red. For each site, the chromosome, genomic coordinates (based on the CRCh38 human reference genome), and gene are also shown.

| **Primary Antibodies** | | | | |
| --- | --- | --- | --- | --- |
| **Target** | **Host/Isotype** | **Clone** | **Catalogue number** | **Dilution** |
| LAMB3 | Rabbit IgG | - | PA5-21514 | 1:100 |
| Cytokeratin 14 | Mouse IgG1 | RCK107 | ab9220 | 1:500 |
| Vimentin | Mouse IgG2a, k | VIM 3B4 | 690013PROGEN | 1:300 |
| Type VII collagen | Mouse IgG1 | LH7.2 | ab6312 | 1:200 |
| p63α | Rat IgG2a, k | W17048F | BIO699501 | 1:100 |
| YAP1 | Rabbit IgG | SU33-06 | MA5-32117 | 1:100 |
| **Secondary Antibodies** | | | | |
| **Target** | **Fluorophore** | **Host/Isotype** | **Catalogue number** | **Dilution** |
| Anti-Rabbit IgG | Alexa Fluor 647 | Goat / IgG | A-21245 | 1:200 |
| Anti-Mouse IgG1 | Alexa Fluor 647 | Goat / IgG | A-21240 | 1:200 |
| Anti-Mouse IgG1 | Alexa Fluor 488 | Goat / IgG | A-21121 | 1:200 |
| Anti-Mouse IgG2a | Alexa Fluor 488 | Goat / IgG | A-21131 | 1:200 |
| Anti-Rat IgG | Alexa Fluor 555 | Goat / IgG | A-21434 | 1:1000 |
| Anti-Rabbit | Alexa Fluor 555 | Goat / IgG | A-21428 | 1:1000 |

**Table S4**. List of primary and secondary antibodies used for immunofluorescence staining.
